# The affinity and selectivity of α‐adrenoceptor antagonists, antidepressants and antipsychotics for the human α2A, α2B, and α2C‐adrenoceptors and comparison with human α1 and β‐adrenoceptors

**DOI:** 10.1002/prp2.936

**Published:** 2022-02-27

**Authors:** Richard G.W. Proudman, Juliana Akinaga, Jillian G. Baker

**Affiliations:** ^1^ Cell Signalling Research Group Division of Physiology, Pharmacology and Neuroscience School of Life Sciences C Floor Medical School Queen’s Medical Centre University of Nottingham Nottingham UK

**Keywords:** affinity, antagonist, antidepressant, antipsychotic, hypertension, selectivity, α‐adrenoceptor

## Abstract

α2‐Adrenoceptors, subdivided into α2A, α2B, and α2C subtypes and expressed in heart, blood vessels, kidney, platelets and brain, are important for blood pressure, sedation, analgesia, and platelet aggregation. Brain α2C‐adrenoceptor blockade has also been suggested to be beneficial for antipsychotic action. However, comparing α2‐adrenoceptor subtype affinity is difficult due to significant species and methodology differences in published studies. Here, ^3^H‐rauwolscine whole cell binding was used to determine the affinity and selectivity of 99 α‐antagonists (including antidepressants and antipsychotics) in CHO cells expressing human α2A, α2B, or α2C‐adrenoceptors, using an identical method to β and α1‐adrenoceptor measurements, thus allowing direct human receptor comparisons. Yohimbine, RX821002, RS79948, and atipamezole are high affinity non‐selective α2‐antagonists. BRL44408 was the most α2A‐selective antagonist, although its α1A‐affinity (81 nM) is only 9‐fold greater than its α2C‐affinity. MK‐912 is the highest‐affinity, most α2C‐selective antagonist (0.15 nM α2C‐affinity) although its α2C‐selectivity is only 13‐fold greater than at α2A. There are no truely α2B‐selective antagonists. A few α‐ligands with significant β‐affinity were detected, for example, naftopidil where its clinical α1A‐affinity is only 3‐fold greater than off‐target β2‐affinity. Antidepressants (except mirtazapine) and first‐generation antipsychotics have higher α1A than α2‐adrenoceptor affinity but poor β‐affinity. Second‐generation antipsychotics varied widely in their α2‐adrenoceptor affinity. Risperidone (9 nM) and paliperidone (14 nM) have the highest α2C‐adrenoceptor affinity however this is only 5‐fold selective over α2A, and both have a higher affinity for α1A (2 nM and 4 nM, respectively). So, despite a century of yohimbine use, and decades of α2‐subtype studies, there remains plenty of scope to develop α2‐subtype selective antagonists.

## Graphical abstract

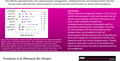

**Abbreviations**
BPH: benign prostatic hyperplasia
CHO: Chinese hamster ovary
sfm: serum free media =DMEM/F12 containing 2mM L‐glutamine

## 1 INTRODUCTION

The α2‐antagonist yohimbine, obtained from the African Corynanthe yohimbe tree (*Pausinystalia johimbe*), has been in clinical use as an aphrodisiac for over a century.^1^, ^2^ It has been used for erectile dysfunction and increases many sexual behaviours through central (CNS) α2‐effects and potential local effects as α2A, α2B, and α2C‐adrenoceptors are expressed in human corpus cavernosum,^1^, ^2^ and can indeed bind yohimbine from tree bark.^3^ The α2‐antagonist idazoxan, developed in 1970s, is selective for α2 over α1‐adrenoceptors, but also binds to other imidazoline binding sites which limits its usefulness in tissue or animal studies.^4^, ^5^ This led to the development of RX821002, a 2‐methyl congener of idazoxan, in the 1980s which retained high α2‐adrenoceptor affinity but without imidazoline receptor affinity (although 5‐HT receptor interactions still occur^6^, ^7^).

α2‐Adrenoceptors are subdivided into α2A, α2B, and α2C‐subtypes. With receptors being present in the heart, blood vessels, and kidney,^8^ α2‐adrenoceptors are important in blood pressure control (an interplay between α1, α2, and β‐adrenoceptors) and including central and peripheral α2‐effects. In addition, many α2‐adrenoceptors present in the brain also have clinical roles in anaesthesia and psychiatric treatments^9^ with both pre‐ and post‐synaptic effects on neurotransmission.^10^, ^11^, ^12^, ^13^

α2A‐adrenoceptors are widely expressed and are important for blood pressure, sedation, analgesia, platelet aggregation, and hypothermia.^14^, ^15^ In the brain, 90% of all α2‐adrenoceptors are of the α2A subtype and they are highly expressed in the prefrontal cortex where activation increases cognitive function.^16^, ^17^ α2A‐adrenoceptor antagonism may be important in sepsis (administration of the α2A‐ antagonist BRL44408 reduced pro‐inflammatory cytokines, TNF‐α and IL‐6 and increased survival in a rat model of sepsis^18^) and potentially clinically relevant α2A‐mirtazapine‐induced reversal of analgesia.^19^ The roles of the α2B‐adrenoceptors are less clear. α2B‐adrenoceptors are involved in blood pressure control (activation causes a hypertensive response related to renal salt balance.^14^ The expression and effects of the α2B‐adrenoceptors appear very minor in the brain.^17^ The α2C‐adrenoceptor is involved in catecholamine release in adrenal chromaffin cells^15^ and in the brain process of startle and stress responses.^14^ α2C‐adrenoceptors form 10% of all brain adrenoceptors but appear particularly prevalent in the striatum and hippocampus.^16^ For certain antipsychotics (e.g., clozapine), α2C‐antagonsim, in addition to dopamine D2 blockade, is thought to be beneficial in the management of schizophrenia^12^, ^13^, ^17^ and α2C‐antagonism may be helpful in improving cognition in dementia.^12^ However, a lack of subtype selective α2‐adrenoceptor ligands has impaired understanding and knowledge of α2‐subtype expression and α2‐subtype function, with much information coming from knockout mice, with subtype adaptation problems that this brings.^12^, ^13^, ^14^, ^15^, ^17^, ^20^

Determining the affinity and selectivity between different α2‐adrenoceptor antagonists has been difficult due to significant variability both within individual, and between different existing studies. Many older studies (pre‐cloned receptors) used different tissue preparations from different species as examples of subtype‐selective tissue, for example, human platelet or cortex for α2A versus neonatal rat lung for α2B.^21^, ^22^, ^23^ However, there are significant species differences. Differences of up to 30‐fold for the affinity of several ligands (including yohimbine and its stereoisomer rauwolscine) for α2A‐adrenoceptors have been reported for human/pig (higher affinity) vs rat/guinea pig (lower affinity).^23^, ^24^, ^25^, ^26^, ^27^, ^28^, ^29^, ^30^, ^31^, ^32^, ^33^, ^34^ Prazosin is the opposite with 15–20‐fold high affinity for rat/mouse kidney receptors than human/rabbit/dog α2A‐adrenoceptors.^4^, ^25^ Overall, it appears that the human α2‐adrenoceptors have more similarity to those of pig, dog, and rabbit than those of rat, mouse, and guinea pig,^6^, ^7^, ^26^, ^27^ which adds further caution with extrapolating from knock‐out mice studies to human clinical relevance of drug actions.

In addition, substantial differences are reported for affinity measurements of single ligands at single subtypes. Reports of prazosin affinity at human α2A‐adrenoceptors range 50‐fold, from 300 nM^21^, ^28^ to a few thousand nM,^23^, ^24^, ^29^ to 16000 nM.^6^ Differences in affinity have also been attributed to technique. A 5‐fold difference in ^3^H‐rauwolscine affinity, and 4‐fold difference in ^3^H‐RX821002 and ^3^H‐atipamezole affinity was found with different buffers.^30^ Thus, previously reported differences in affinity are likely to be due to several explanations: species is very important but techniques (cloned receptor vs. whole tissue, membrane vs. whole cell, different buffers) are also important and make direct comparison of studies difficult.

This study therefore measured the affinity and selectivity of a wide range of α‐antagonists (including antidepressants and antipsychotics) in living CHO cells expressing the human α2A, α2B, or α2C‐adrenoceptor. Furthermore, as these measurements were determined using an identical technique in human β1 and β2‐adrenoceptors (included here, and^31^, ^32^) and α1‐adrenoceptors,^33^ this study explores the affinity and selectivity of ligands across the human adrenoceptors commonly targeted for cardiovascular, urological and CNS effects.

## 2 MATERIALS AND METHODS

### 2.1 Materials

All compounds, together with the supplier and catalogue number are given in alphabetical order in Supplementary Data Table 1. White sided view plates were from Greiner Bio‐one, Kremsmunster, Austria. ^3^H‐rauwolscine (a stereoisomer of yohimbine, specific activity 82.9), ^3^H‐RX821002 (specific activity 36.5), ^3^H‐CGP12177 (specific activity 37.7), Microscint 20 and Ultima Gold XL scintillation fluid were from PerkinElmer (Buckinghamshire, UK). Foetal calf serum was from Gibco (Thermo‐Fisher), Lipofectamine and OPTIMEM were from Life Technologies, Thermo‐Fisher, Massachusetts USA. All other cell culture reagents were from Sigma Chemicals (Poole, Dorset, UK).

**TABLE 1:** Log K_D_ values obtained from inhibition of ^3^H‐RX821002 or ^3^H‐rauwolscine binding to the human α2A, α2B, and α2C‐adrenoceptors in living cells. Values represent mean ±s.e.mean of n separate experiments. Compounds are arranged in order of α2A‐affinity.

	^3^H‐RX821002 as radioligand						^3^H‐rauwolscine as radioligand					
	Log K_D_ α2A		Log K_D_ α2B		Log K_D_ α2C		Log K_D_ α2A		Log K_D_ α2B		Log K_D_ α2C	
MK912	−8.76 ± 0.05	5	−8.23 ± 0.11	5	−10.00 ± 0.15	4	−8.71 ± 0.05	8	−8.16 ± 0.10	8	−9.82 ± 0.11	9
yohimbine	−8.58 ± 0.03	5	−7.66 ± 0.05	5	−8.78 ± 0.10	5	−8.48 ± 0.07	5	−7.66 ± 0.10	5	−8.52 ± 0.05	5
RX821002	−8.23 ± 0.02	5	−7.67 ± 0.04	5	−8.28 ± 0.07	5	−8.10 ± 0.07	5	−7.45 ± 0.06	5	−8.14 ± 0.02	5
WB4101	−7.58 ± 0.05	6	−6.88 ± 0.05	6	−8.24 ± 0.13	6	−7.55 ± 0.05	6	−6.77 ± 0.05	6	−8.17 ± 0.05	6
BRL44408	−7.24 ± 0.05	6	−5.59 ± 0.05	6	−6.32 ± 0.09	6	−7.19 ± 0.04	7	−5.41 ± 0.04	7	−6.22 ± 0.07	7
carvedilol	−6.58 ± 0.04	5	−6.46 ± 0.05	5	−7.46 ± 0.14	5	−6.54 ± 0.02	5	−6.31 ± 0.02	5	−7.32 ± 0.05	5
ARC239	−5.99 ± 0.06	4	−7.29 ± 0.14	4	−7.18 ± 0.05	4	−5.99 ± 0.06	5	−7.32 ± 0.14	6	−7.25 ± 0.14	5
chlorpromazine	−5.57 ± 0.11^app^	6	−6.63 ± 0.11	6	−6.02 ± 0.16	6	−5.65 ± 0.13^app^	6	−6.60 ± 0.12	6	−5.93 ± 0.11	6
prazosin	−5.41 ± 0.03	6	−6.34 ± 0.03	6	−6.48 ± 0.09	6	−5.33 ± 0.05	6	−6.17 ± 0.05	6	−6.59 ± 0.04	6
JP1302	−5.22 ± 0.04	5	−5.22 ± 0.04	5	−6.57 ± 0.26	5	−5.29 ± 0.04	5	−5.11 ± 0.05	5	−6.92 ± 0.13	5
labetalol	−4.63 ± 0.04^app^	5	−4.99 ± 0.07^app^	5	−5.42 ± 0.05	5	−4.62 ± 0.07^app^	5	−4.71 ± 0.08^app^	5	−5.27 ± 0.04	5

Note

^apparent^ the maximum concentration of competing ligand inhibited most but not all of specific binding. An IC_50_ was determined by extrapolating the curve assuming that all specific binding would be inhibited if a higher concentration of competing ligand were possible. Thus an apparent K_D_ was calculated.

### 2.2 Cell lines

CHO‐K1 (RIDD: CVCL_0214) were stably transfected with the human α2A‐adrenoceptor, human α2B‐adrenoceptor or human α2C‐adrenoceptor DNA (DNAs from Guthrie DNA Resource Centre) using Lipofectaime and Optimem according to the manufacturer’s instructions. Following 3 weeks selection using resistance to neomycin (at 1 mg/ml), single clones from each transfection were isolated by dilution cloning. Thus stable cell lines CHO‐α2A, CHO‐α2B, and CHO‐α2C were created. CHO lines stable expressing the human β1 or β2‐adrenoceptor were also used.^31^

### 2.3 Cell culture

CHO cells were grown in Dulbecco’s modified Eagle’s medium nutrient mix F12 (DMEM/F12) containing 10% foetal calf serum and 2mM L‐glutamine in a 37°C humidified 5% CO_2_: 95% air atmosphere. Cells were seeded into white‐sided, clear bottomed 96‐well view plates and grown to confluence. Cells were always grown in the absence of any antibiotics. Mycoplasma contamination has intermittently been monitored within the laboratory (negative) but cell lines were not tested routinely with each experiment.

### 2.4 ^3^H‐rauwolscine and ^3^H‐RX821002 whole cell saturation binding

The K_D_ value for both radioligands was determined in each cell line by saturation binding. The radioligands were diluted to twice the final concentration in serum‐free media (sfm, DMEM/F12 containing 2 mM L‐glutamine). Media was removed from each well and replaced with either 100 µl sfm (total binding) or 100 µl, 20 µM RX821002 (when ^3^H‐rauwolscine used) or 20 μM yohimbine (when ^3^H‐RX821002 used) in sfm to determine non‐specific binding. 100µl radioligand was then added to the wells (quadruplicates per condition =1 in 2 dilution in well), and the plates incubated at 37°C (humidified 5% CO_2_: 95% air atmosphere) for 2 h. After 2 h, the cells were washed twice by the addition and removal of 2×200 µl cold (4°C) phosphate‐buffered saline. A white base was applied to the plate to convert the wells into white‐sided/white‐bottomed plates, 100μl Microscint 20 was added to each well and a transparent top seal applied to the plates. Plates were left at room temperature in the dark for at least 6 h before being counted on a Topcount (PerkinElmer, 2‐min count per well).

### 2.5 ^3^H‐rauwolscine, ^3^H‐RX821002, and ^3^H‐CGP12177 whole cell competition binding

Affinity was assessed using the whole cell binding method of.^31^ Ligands were diluted in sfm to twice their final concentration. Media was removed from each well and 100µl ligand added to triplicate wells. This was immediately followed by the addition of 100µl radioligand (diluted in sfm) and the cells incubated for 2 h at 37°C (5% CO_2_, humidified atmosphere), after which the plates were washed as above. Cells were inspected under a light microscope to ensure cells were still adherent after the wash and before the addition of Microscint 20. In a few cases, high concentrations of competing ligand caused the cells to round up and be washed off the plates. These concentrations were excluded from the analysis. Total binding (6 wells/plate) and non‐specific binding (6 wells/plate (determined by the presence of 10µM yohimbine or 10µM RX821002 in sfm) was defined in every plate.

Given the two‐component inhibition of ^3^H‐prazosin binding seen with dibenamine and phenoxybenzamine at the α1‐adrenoceptors, sodium thiosulphate, which reacts with the ethyleniminium ions, was used in dibenamine and phenoxybenzamine experiments, in excess, as in Ref.^33^

Thus all studies in human β, α1, and α2‐adrenoceptors have been conducted in intact living mammalian cells using the same method. The only differences between the experiments are the radioligand, the ligand used to define non‐specific binding and the transfected receptor. As all experiments were conducted in living cells, physiological levels of intracellular endogenous GTP will always have been present and potentially are therefore more akin to how drugs bind in people, rather than studies conducted in membrane preparations. There is theoretically a potential difference in affinity measurement if compounds have a different intrinsic efficacy for different receptor subtypes. Thus, if one compound is a partial agonist at one receptor subtype but an inverse agonist at another, a different receptor state is induced upon binding to the receptor. This may therefore affect how the compound and radioligand compete for the receptor, which in turn could theoretically affect affinity measurements. As this study was aimed at studying antagonists, this effect is likely to be minimal.

### 2.6 Data analysis

Saturation curves for specific radioligand binding were plotted using the following equation in GraphPad Prism 7:

$$\text{Specific}\;\text{binding}=B_{\text{max}}\times K_{D}\left(\left[{}^{3}H‐\text{radioligand}\right]+K_{D}\right)$$

where B_max_ is the maximum specific binding, K_D_ is the dissociation constant of the radioligand and [^3^H‐radioligand] is the concentration of the radioligand.

In all cases where a K_D_ value is stated, increasing concentrations of the competing ligand fully inhibited the specific binding of the radioligand (unless otherwise annotated in the tables). The following equation was then fitted to the data using Graphpad Prism 7 and the IC_50_ was then determined as the concentration required to inhibit 50% of the specific binding.

$$\%\text{Specific}\;\text{binding}=100‐\left(100\times \left[A\right]/\left(\left[A\right]+{\text{IC}}_{50}\right)\right)$$

where [A] is the concentration of the competing ligand and IC_50_ is the concentration at which half of the specific binding of radioligand that has been inhibited.

From the IC_50_ value, the known concentration of radioligand and the known radioligand K_D_ for at each receptor, a K_D_ (concentration at which half the receptors are bound by the competing ligand) value was calculated using the Cheng‐Prusoff equation:

$$K_{D}\;\text{competing ligand}=\frac{{\text{IC}}_{50}}{1+\left(\left[{}^{3}H‐\text{radioligand}\right]/K_{D}{}^{3}H‐\text{radioligand}\right)}$$

In some cases, the maximum concentration of competing ligand was not able to inhibit all of the specific binding. Where no inhibition of radioligand binding was seen, even with a maximum concentration of competing ligand possible, “no binding” is given in the tables. Where the inhibition produced by the maximum concentration of the competing ligand was 50% or less, an IC_50_ could not be determined and thus a K_D_ value not calculated. This is shown in the tables as IC_50_>top concentration used (i.e., IC_50_>100µM means that 100µM inhibited some but less than 50% of the specific binding). In cases where the competing ligand caused a substantial (greater than 50%, but not 100%) inhibition of specific binding, an IC_50_ value was determined by extrapolating the curve to non‐specific levels and assuming that a greater concentration would have resulted in 100% inhibition. These values are given as apparent K_D_ values in the tables.

For some ligands, a one‐component sigmoidal fit was visually not a good fit for the inhibition of ^3^H‐rauwolscine binding (e.g., Figure 2B) in which case a two‐component curve was used, using the equation below:

$$\%\;\text{specific}\;\text{binding}\;=\frac{[A].N}{\left([A]+{\text{IC}}_{50}1\right)}+\frac{\left[A].(100‐N\right)}{\left([A]+\;{\text{IC}}_{50}2\right)}.$$

where [A] is the concentration of the competing ligand, IC_50_1 and IC_50_2 are the respective IC_50_ values for the two components and N is the percentage of the response occurring through the first component (IC_50_1). K_D_ values were calculated from IC_50_ values as above.

Radioligand concentrations were determined from taking the average of triplicate 50µl samples of each radioligand concentration used and counted on a PerkinElmer Scintillation counter.

Selectivity ratios are given as a ratio of the K_D_ values for the different receptors.

In view of the higher level of receptor expression in these cell lines and concerns about depletion of the free radioligand in the binding assays, depletion was monitored. Free radioligand depletion of 20% was encountered (resulting in a potential inaccuracy of 0.04 log units in the stated K_D_ values). Ligand depletion of a maximum of 25–33% were noted in occasional experiments. This results in a potential inaccuracy of 0.06 to 0.08 log units in the stated K_D_ value of the competing ligands. However, as radioligand depletion would not have been constant through the displacement curve, with only half the depletion at IC_50_ (i.e., usually therefore an error of 0.02 log units for the calculated K_D_ value, or up to 0.04 log units in the worst cases), this is within experimental error and does not substantially affect the results. Data are therefore plotted and K_D_ values calculated assuming no radioligand depletion.

Nomenclature of Targets and Ligands.

Key protein targets and ligands in this article are hyperlinked to corresponding entries in http://www.guidetopharmacology.org, the common portal for data from the IUPHAR/BPS Guide to PHARMACOLOGY,^34^ and are permanently archived in the Concise Guide to PHARMACOLOGY 2019/20.^35^

## 3 RESULTS

### 3.1 Evaluation of ^3^H‐rauwolscine and ^3^H‐RX821002 for whole cell binding

^3^H‐rauwolscine and ^3^H‐RX821002 have previously been used for membrane binding studies in both cell lines and with human tissue (e.g.,.^20^, ^21^, ^30^, ^36^, ^37^). However, given the reported differences in off target affinity, both radioligands were investigated for their suitability for studying radioligand binding in whole living cells. Saturation binding yielded a K_D_ value for ^3^H‐rauwolscine in CHO‐α2A cell of 2.79 ± 0.24 nM (5830 ± 853 fmol/mg protein, n=7), in CHO‐α2B cells of 7.87 ± 0.78 nM (13102 ± 2805 fmol/mg protein, n=9) and in CHO‐α2C cells of 0.76 ± 0.07 nM (1379 ± 98 fmol/mg protein, n = 9). For ^3^H‐RX821002 saturation‐binding studies, the values were K_D_ 4.73 ± 0.42 nM (4584 ± 667 fmol/mg protein, n=8) in CHO‐α2A cells, 17.96 ± 1.41 nM (11326 ± 3531 fmol/mg protein, n = 6) in CHO‐α2B cells and 3.60 ± 0.24 nM (798 ± 143 fmol/mg protein, n=6) in CHO‐α2C cells. Several ligands were investigated in competition studies using both radioligands and very similar results were obtained (Table 1). Thus both ^3^H‐rauwolscine and ^3^H‐RX821002 are good ligands for whole cell studies in living CHO cells with transfected human α2‐adrenoceptors. ^3^H‐rauwolscine was chosen for all further studies as its affinity was slightly higher at all three receptors.

### 3.2 Affinity and selectivity of ligands at α2‐adrenoceptors

**FIGURE 1:**
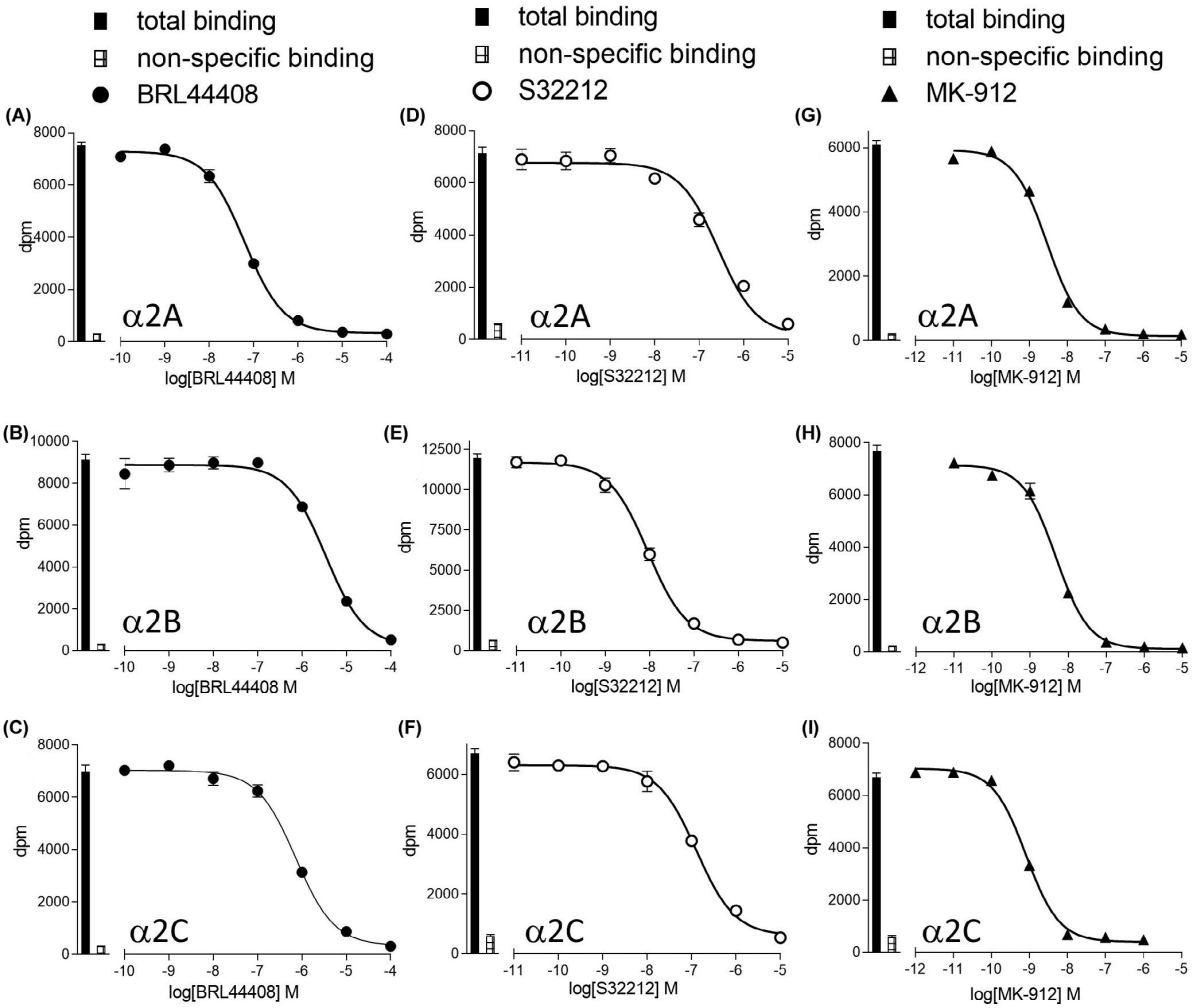
Inhibition of ^3^H‐rauwolscine binding to whole cells by BRL44408 (A–C), S32212 (D–F) or MK‐912 (G–I) to CHO‐α2A cells (A, D, G), CHO‐α2B cells (B, E, H) or CHO‐α2C cells (C, F, I). Bars represent total ^3^H‐rauwolscine and non‐specific binding (determined in the presence of 10μM RX821002. The concentration of ^3^H‐rauwolscine was (A) 0.99 nM, (B) 0.99 nM, (C) 0.99 nM, (D) 0.88 nM, (E) 0.88 nM, (F) 0.88 nM, (G) 0.86 nM, (H) 0.86 nM, and (I) 0.88 nM. Data points are mean ±s.e.mean of triplicate determinations

**FIGURE 2:**
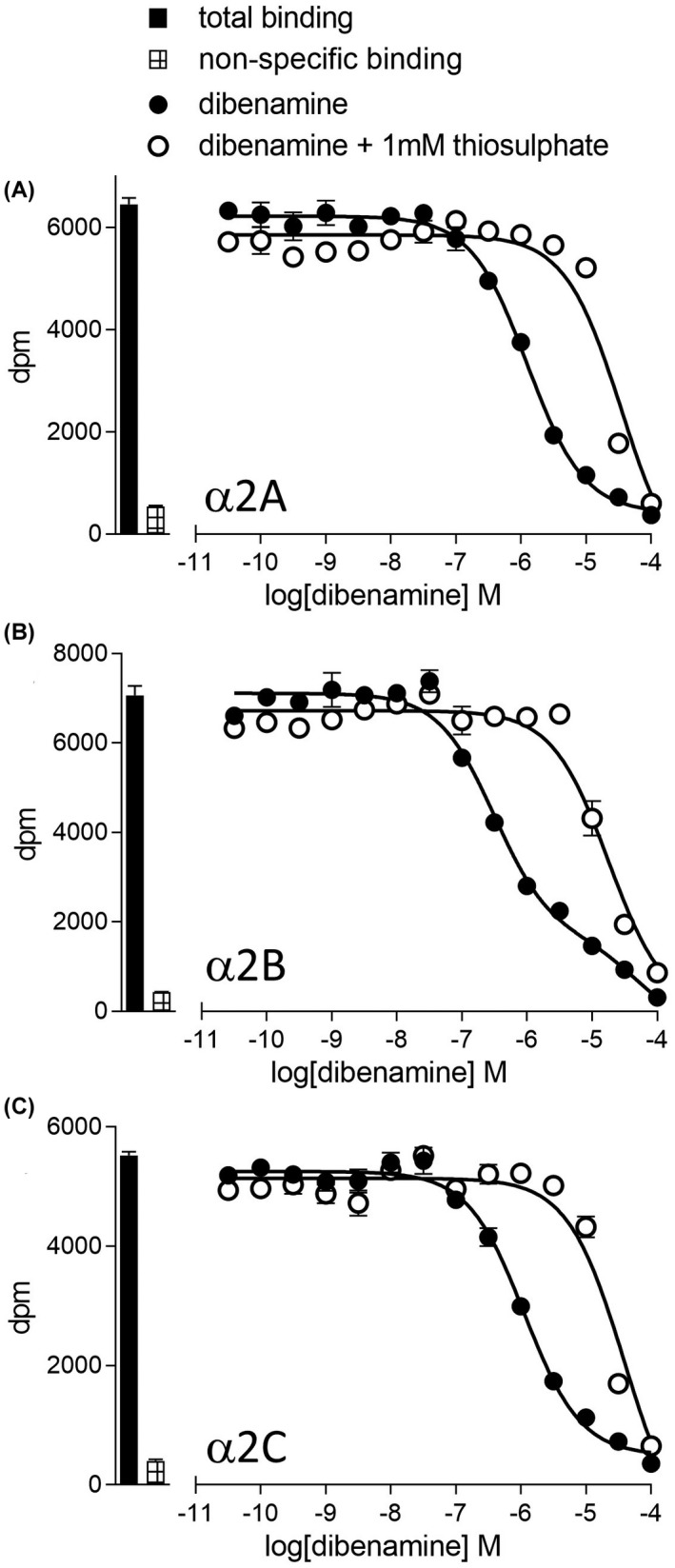
Inhibition of ^3^H‐rauwolscine binding to whole cells by dibenamine following pre‐incubation of dibenamine with sfm or 1mM thiosulphate to CHO‐α2A cells (A), CHO‐α2B cells (B), or CHO‐α2C cells (C). Bars represent total ^3^H‐rauwolscine binding and non‐specific binding as determined in the presence of 10 μM RX821002. The concentration of ^3^H‐rauwolscine was 0.74 nM in all cases. Data points are mean ±s.e.mean of triplicate determinations.

The affinity and selectivity of a large range of α‐adrenoceptor antagonists was evaluated (Figure 1; Table 2). It is clear that there are few α2‐subtype selective ligands. Dibenamine and phenoxybenzamine inhibited ^3^H‐rauwolscine binding in a manner best described by a two‐component response in CHO‐α2B cells for both compounds and for phenoxybenzamine in CHO‐α2C cells (Figure 2, Table 2) in a manner similar to that seen in the α1‐adrenoceptors.^33^ The responses in CHO‐α2A cells and for dibenamine in CHO‐α2C cells were too low affinity for a second component to be clearly determined. Dibenamine and phenoxybenzamine both contain a nitrogen mustard group, which cyclises to form ethyleniminium ions.^38^ Sodium thiosulphate reacts with the ethyleniminium ions preventing them interacting with α‐adrenoceptors.^38^ Preincubation with sodium thiosulphate abolished the higher affinity components and reduced the affinity of both ligands at all three receptors a follows: dibenamine −4.59 ± 0.08 n=5, −4.64 ± 0.07 n=5, and −4.64 ± 0.11 n = 5 for α2A, α2B, and α2C, respectively; and for phenoxybenzamine −4.71 ± 0.13 n = 5, −4.86 ± 0.08 n = 5, and −4.96 ± 0.10 n = 5 for α2A, α2B, and α2C, respectively and are therefore similar to the second component response. The higher affinity K_D_ values in Table 2 are therefore highly likely to be the affinity of the ligand interacting with the receptor (as in^33^).

**TABLE 2:** Log K_D_ values obtained from inhibition of ^3^H‐rauwolscine binding by adrenoceptor antagonists to the human α2A, α2B, and α2C‐adrenoceptors in living cells. Values represent mean ±s.e.mean of n separate experiments. Selectivity ratios are also given where a ratio of 1 demonstrates no selectivity for a given receptor subtype over another. Thus BRL44408 has 60‐fold higher affinity for the α2A than the α2B‐adrenoceptor. Compounds are arranged in order of α2A‐selectivity.

Ligand	Affinity measurements						Selectivity ratios					
	Log K_D_ α2A	n	Log K_D_ α2B	n	Log K_D_ α2C	n	α2A vs α2B		α2A vs α2C		α2B vs α2C	
BRL 44408	−7.19 ± 0.04	7	−5.41 ± 0.04	7	−6.22 ± 0.07	7	60.3		9.3			6.5
benoxathian	−7.17 ± 0.02	5	−5.96 ± 0.06	5	−7.75 ± 0.03	5	16.2			3.8		61.7
tamsulosin	−6.33 ± 0.04	5	−5.31 ± 0.04	5	−6.41 ± 0.03	5	10.5			1.2		12.6
alfuzosin	−5.56 ± 0.04	5	−4.62 ± 0.05	5	−6.14 ± 0.04	5	8.7			3.8		33.1
2‐MPMDQ	−6.79 ± 0.04	5	−5.94 ± 0.09	5	−7.50 ± 0.02	5	7.1			5.1		36.3
yohimbine	−8.48 ± 0.07	5	−7.66 ± 0.10	5	−8.52 ± 0.05	5	6.6			1.1		7.2
idazoxan	−7.17 ± 0.04	5	−6.39 ± 0.05	5	−7.16 ± 0.03	5	6.0		1.0			5.9
WB4104	−7.55 ± 0.05	6	−6.77 ± 0.05	6	−8.17 ± 0.05	6	6.0			4.2		25.1
A80426	−7.24 ± 0.08	6	−6.52 ± 0.06	6	−7.46 ± 0.07	6	5.2			1.7		8.7
eforaxan	−7.58 ± 0.05	5	−6.88 ± 0.07	5	−7.44 ± 0.04	5	5.0		1.4			3.6
2‐PMDQ	−6.83 ± 0.05	5	−6.14 ± 0.08	5	−7.07 ± 0.02	5	4.9			1.7		8.5
atipamezole	−8.50 ± 0.08	5	−7.85 ± 0.04	5	−8.48 ± 0.09	5	4.5		1.0			4.3
RX 821002	−8.10 ± 0.07	5	−7.45 ± 0.06	5	−8.14 ± 0.02	5	4.5			1.1		4.9
sunepitron	−7.28 ± 0.04	6	−6.65 ± 0.08	6	−8.11 ± 0.04	6	4.3			6.8		28.8
doxazosin	−5.35 ± 0.04	6	−4.74 ± 0.07^app^	6	−6.24 ± 0.02	6	4.1			7.8		31.6
phentolamine	−7.26 ± 0.03	5	−6.69 ± 0.05	5	−6.92 ± 0.04	5	3.7		2.2			1.7
MK−912	−8.71 ± 0.05	8	−8.16 ± 0.10	8	−9.82 ± 0.11	9	3.6			12.9		45.7
RS17053	−6.20 ± 0.11	5	−5.65 ± 0.07	5	−6.35 ± 0.08	5	3.5			1.4		5.0
RS100329	−7.00 ± 0.03	5	−6.47 ± 0.04	5	−7.82 ± 0.03	5	3.4			6.6		22.4
lisuride	−8.99 ± 0.05	5	−8.52 ± 0.05	5	−9.27 ± 0.05	5	3.0			1.9		5.6
BMY7378	−5.30 ± 0.03	5	−4.98 ± 0.09^app^	5	−6.26 ± 0.01	5	2.1			9.1		19.1
RS79948	−8.93 ± 0.03	5	−8.57 ± 0.03	5	−9.36 ± 0.04	5	2.3			2.7		6.2
carvedilol	−6.54 ± 0.02	5	−6.31 ± 0.02	5	−7.32 ± 0.05	5	1.7			6.0		10.2
JP1302	−5.29 ± 0.04	5	−5.11 ± 0.05	5	−6.92 ± 0.13	5	1.5			42.7		64.6
SKF86466	−6.29 ± 0.05	5	−6.17 ± 0.047	5	−6.39 ± 0.04	5	1.3			1.3		1.7
3‐MPPI	−6.67 ± 0.05^ep^	5	IC_50_>−4	5	−7.01 ± 0.03^ep^	5				2.2		
PF3774076	−5.59 ± 0.04	6	IC_50_>−4	6	−5.29 ± 0.09	6			2.0			
Rec15‐2615	−5.53 ± 0.12^app^	6	IC_50_>−4.5	6	−6.56 ± 0.13	6				10.7		
AH11110A	−4.70 ± 0.04^app^	5	IC_50_>−4	5	−4.86 ± 0.03^app^	5				1.4		
silodosin	−5.49 ± 0.06^app^	6	IC_50_>−5	6	−6.12 ± 0.06^app^	6				4.3		
5‐methyl‐urapidil	−5.18 ± 0.05	5	−5.17 ± 0.05	5	−5.81 ± 0.07	5	1.0			4.3		4.4
SNAP5089	IC_50_>−5	5	IC_50_>−5	5	−5.65 ± 0.06	5						
anisodamine	IC_50_>−3	5	IC_50_>−3	5	−3.56 ± 0.07^app^	5						
2‐niguldipine	IC_50_>−5	5	−5.48 ± 0.11	5	−6.07 ± 0.11	5						3.9
naftapidil	−6.55 ± 0.09	5	−6.60 ± 0.07	5	−7.17 ± 0.08	5		1.1		4.2		3.7
labetolol	−4.62 ± 0.07^app^	5	−4.71 ± 0.08^app^	5	−5.27 ± 0.04	5		1.2		4.5		3.6
ifenprodil	−6.01 ± 0.05	5	−6.14 ± 0.06	5	−6.80 ± 0.05	5		1.3		6.2		4.6
domperidone	−5.09 ± 0.06^app^	6	−5.29 ± 0.07	6	−5.78 ± 0.08	6		1.6		4.9		3.1
urapidil	−5.49 ± 0.05	5	−5.78 ± 0.08	5	−6.34 ± 0.05	5		1.9		7.1		3.6
HEAT	−7.45 ± 0.04	5	−7.72 ± 0.11	5	−8.05 ± 0.19	5		1.9		4.0		2.1
indoramin	−5.13 ± 0.03^app^	6	−5.46 ± 0.05	6	−5.80 ± 0.05	6		2.1		4.7		2.2
cyclazosin	−5.00 ± 0.03	5	−5.35 ± 0.13	5	−6.18 ± 0.02	5		2.2		15.1		6.8
imiloxan	−5.88 ± 0.03	6	−6.48 ± 0.05	6	−6.27 ± 0.03	6		4.0		2.5	1.6	
dibenamine	−5.80 ± 0.06	10	−6.43 ± 0.06 −4.64 ± 0.07 60.9 ± 3.4% site 1	10	−6.18 ± 0.05	10		4.3		2.4	1.8	
promethazine	−5.58 ± 0.07	5	−6.25 ± 0.06	5	−5.54 ± 0.05	5		4.7	1.1		5.1	
phenoxybenzamine	−5.72 ± 0.10	10	−6.44 ± 0.11 −4.89 ± 0.08 51.4 ± 3.3% site 1	10	−6.41 ± 0.11 −4.71 ± 0.13 74.1 ± 4.1% site 1	10		5.2		4.9	1.1	
prazosin	−5.33 ± 0.05	6	−6.17 ± 0.05	6	−6.59 ± 0.04	6		6.9		18.2		2.6
terazosin	−5.18 ± 0.03	5	−6.08 ± 0.05	5	−6.27 ± 0.08	5		7.9		12.3		1.5
spiroxatrine	−6.97 ± 0.03	6	−7.87 ± 0.07	6	−8.74 ± 0.04	6		7.9		58.9		7.4
S32212	−6.62 ± 0.13	8	−7.80 ± 0.10	8	−7.18 ± 0.10	8		15.1		3.6	4.2	
ARC239	−5.99 ± 0.06	5	−7.32 ± 0.14	6	−7.25 ± 0.14	5		21.4		18.2	1.2	
β‐blockers												
cyanopindolol	−5.56 ± 0.10	5	−4.82 ± 0.10^app^	5	−6.15 ± 0.07	5	5.5			3.9		21.4
bucindolol	−5.81 ± 0.05	5	−5.63 ± 0.06	5	−5.95 ± 0.04	5	1.5			1.4		2.1
ICI118551	−5.03 ± 0.03	5	IC_50_>−4	5	−5.05 ± 0.04	5			1.0			
SDZ21009	−4.86 ± 0.07^app^	6	IC_50_>−4	6	IC_50_>−4.5	6						
propranolol	−4.85 ± 0.02	5	IC_50_>−4	5	−4.71 ± 0.06	5			1.4			
carazolol	−4.66 ± 0.06^app^	6	IC_50_>−4	6	−4.66 ± 0.05^app^	6			1.0			
CGP12177	IC_50_>−3	5	No bind to 1mM	5	IC_50_>−3	5						
CGP20712A	IC_50_>−4	5	IC_50_>−4	5	−5.17 ± 0.03	5						

Note

^app^ = apparent affinity. The maximum concentration of competing ligand inhibited most but not all of specific binding. An IC_50_ was determined by extrapolating the curve assuming that all specific binding would be inhibited if a higher concentration of competing ligand were possible. Thus an apparent K_D_ was calculated.

^ep^ = early plateau, the competing ligand did not fully inhibit specific binding and the inhibition curve reached a plateau of maximal inhibition of binding. The specific binding inhibited by 3‐MPPI was 75.6 ± 0.9% at α2A and 87.1 ± 1.5% at α2C

**FIGURE 3:**
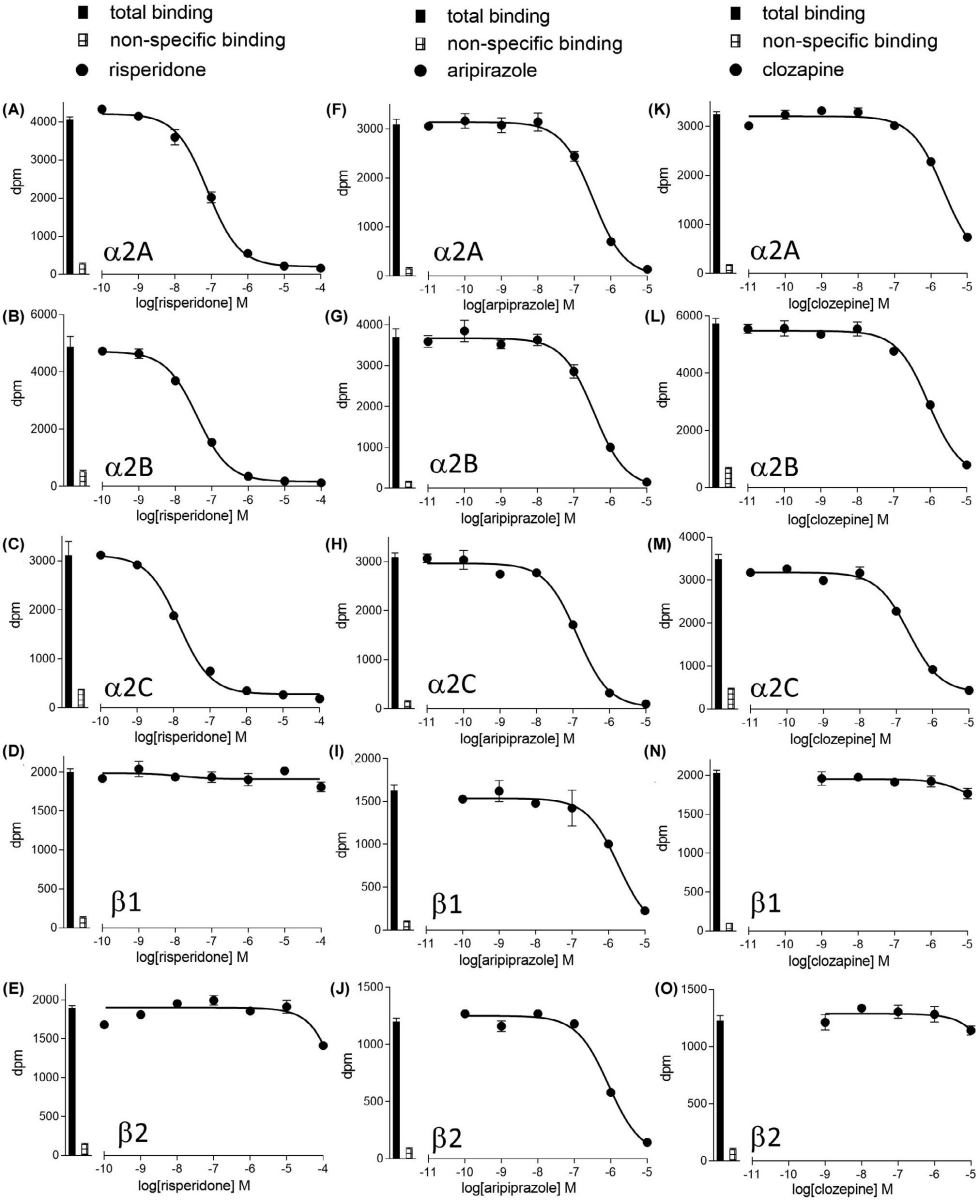
Inhibition of ^3^H‐rauwolscine (α2A, α2B, and α2C) or ^3^H‐CGP12177 (β1 and β2) binding to whole cells by (A–E) risperidone, (F–J) aripiprazole and (K–O) clozapine to CHO‐α2A cells, CHO‐α2B cells, CHO‐α2C cells, CHO‐β1 cells, CHO‐β2 cells. Bars represent total radioligand binding and non‐specific binding as determined in the presence of 10μM RX821002 (α2A, α2B, and α2C cells) or 10μM propranolol (β1 and β2 cells). The concentration of radioligand was (A) 0.54 nM, (B) 0.54 nM, (C) 0.54 nM, (D) 0.77 nM, (E) 1.00 nM, (F) 0.50 nM, (G) 0.50 nM, (H) 0.50 nM, (I) 0.72 nM, (J) 0.72 nM, (K) 0.50 nM, (L) 0.54 nM, (M) 0.54 nM, (N) 0.94 nM and (O) 0.72 nM. Data points are mean ±s.e.mean of triplicate determinations

Given the more recent suggestions of α2C affinity being important for antipsychotic drug actions, the affinity and selectivity of antidepressants (Table 3) and antipsychotics (Figure 3; Table 4) were examined.

**TABLE 3:** Log K_D_ values of antidepressants binding to the human α2A, α2B and α2C‐adrenoceptors. Values represent mean ±s.e.mean of n separate experiments. Selectivity ratios are also given, where a ratio of 1 demonstrates no selectivity for a given receptor subtype over another. Thus, clompiramine has 2.5‐fold higher affinity for the α2B than the α2A‐adrenoceptor. Compounds are arranged in order of α2A‐selectivity.

ligand	Affinity measurements						Selectivity ratios					
	Log K_D_ α2A	n	Log K_D_ α2B	n	Log K_D_ α2C	n	α2A vs α2B		α2A vs α2C		α2B vs α2C	
Tricyclic antidepressants												
clomipramine	−5.71 ± 0.07^app^	5	−6.10 ± 0.13	5	−5.80 ± 0.02^app^	5		2.5		1.2	2.0	
protriptyline	−5.00 ± 0.05	5	−5.39 ± 0.13	5	−5.26 ± 0.07	5		2.5		1.8	1.3	
norclomipramine	−5.29 ± 0.09^app^	6	−5.74 ± 0.04^app^	6	−5.80 ± 0.07^app^	6		2.8		3.2		1.1
trimipramine	−5.67 ± 0.03	5	−6.22 ± 0.05	5	−6.37 ± 0.03	5		3.5		5.0		1.4
nortriptyline	−5.65 ± 0.05	5	−6.38 ± 0.02	5	−6.19 ± 0.08	5		5.4		3.5	1.5	
desipramine	−5.04 ± 0.06	5	−5.78 ± 0.04	5	−5.52 ± 0.03	5		5.5		3.0	1.8	
lofepramine	−4.86 ± 0.04^app^	5	−5.60 ± 0.08	5	−5.28 ± 0.06	5		5.5		2.6	2.1	
doxepin	−5.69 ± 0.12	5	−6.67 ± 0.05	5	−6.04 ± 0.07	5		9.5		2.2	4.3	
dosulepin	−5.16 ± 0.06	5	−6.20 ± 0.06	5	−5.63 ± 0.11	5		11.0		3.0	3.7	
imipramine	−5.25 ± 0.04	5	−6.36 ± 0.08	5	−5.89 ± 0.03	5		12.9		4.4	3.0	
amitriptyline	−5.86 ± 0.05^app^	5	−7.12 ± 0.05	5	−6.67 ± 0.09	5		18.2		6.5	2.8	
Tetracyclic antidepressants												
mirtazepine	−6.80 ± 0.05	5	−6.09 ± 0.06	5	−6.96 ± 0.03	5	5.1			1.4		7.4
other noradrenaline and serotonin reuptake inhibitors												
duloxetine	−5.43 ± 0.06	5	−5.31 ± 0.09	5	−5.67 ± 0.06	5	1.3			1.7		2.3
venlafaxime	−3.46 ± 0.03^app^	5	IC_50_>−3	5	−3.74 ± 0.11^app^	5				1.9		
Noradrenaline reuptake inhibitors												
reboxetine	IC_50_>−4	5	IC_50_>−4	5	−4.56 ± 0.07^app^	4						
Selective serotonin reuptake inhibitors (SSRI)												
fluvoxamine	−4.81 ± 0.04^app^	6	−4.37 ± 0.08app	5	−4.82 ± 0.07^app^	6	2.8		1.0			2.8
sertraline	−5.67 ± 0.07^app^	6	−5.62 ± 0.11^app^	6	−5.64 ± 0.05^app^	6	1.1		1.1		1.0	
fluoxetine	−4.70 ± 0.10^app^	5	−4.99 ± 0.03	5	−4.79 ± 0.07^app^	5		1.9		1.2	1.6	
citalopram	IC_50_>−4	5	IC_50_>−4	5	IC_50_>−4	5						
paroxetine	IC_50_>−5	5	IC_50_>−5	5	IC_50_>−5	5						
Serotonin reuptake inhibitors												
vortioxetine	−5.63 ± 0.06^app^	5	−5.32 ± 0.04^app^	6	−5.84 ± 0.05	6	2.0			1.6		3.3
trazodone	−6.17 ± 0.08	5	−5.96 ± 0.07	5	−6.69 ± 0.04	5	1.6			3.3		5.4

Note

^app^ = apparent affinity The maximum concentration of competing ligand inhibited most but not all of specific binding. An IC_50_ was determined by extrapolating the curve assuming that all specific binding would be inhibited if a higher concentration of competing ligand were possible.

**TABLE 4:** Log K_D_ values of antipsychotics binding to the human α2A, α2B, and α2C‐adrenoceptors. Values represent mean ±s.e.mean of n separate experiments. Selectivity ratios are also given where a ratio of 1 demonstrates no selectivity for a given receptor subtype over another. Compounds are arranged in order of α2A‐selectivity.

Ligand	Affinity measurements						Selectivity ratios					
	Log K_D_ α2A	n	Log K_D_ α2B	n	Log K_D_ α2C	n	α2A vs α2B		α2A vs α2C		α2B vs α2C	
First‐generation antipsychotics												
sulpiride	−4.50 ± 0.02	5	−4.37 ± 0.06	5	−4.67 ± 0.07	5	1.3			1.5		2.0
haloperidol	−5.38 ± 0.06	5	−5.53 ± 0.10	5	−5.77 ± 0.05	5		1.4		2.5		1.7
flupenthixol	−6.10 ± 0.12	5	−6.28 ± 0.13	5	−6.88 ± 0.14	5		1.5		6.0		4.0
pimozide	−5.76 ± 0.12^ep^	5	−6.30 ± 0.10	5	−6.84 ± 0.05	5		3.5		12.0		3.5
trifluoperazine	−5.60 ± 0.05	5	−6.22 ± 0.12	5	−6.20 ± 0.06	5		4.2		4.0	1.0	
prochlorperazine	−5.78 ± 0.02^app^	6	−6.46 ± 0.11	6	−6.31 ± 0.09	6		4.8		3.4	1.4	
chlorpromazine	−5.65 ± 0.13^app^	6	−6.60 ± 0.12	6	−5.93 ± 0.11	6		8.9		1.9	4.7	
perphenazine	−6.00 ± 0.06	6	−7.16 ± 0.05	6	−6.83 ± 0.04	5		14.5		6.8	2.1	
Second‐generation antipsychotics												
amisulpiride	−5.11 ± 0.09^app^	5	−4.69 ± 0.13^app^	5	−5.57 ± 0.07	5	2.6			2.9		7.6
aripirazole	−6.68 ± 0.08	5	−6.54 ± 0.08	6	−7.23 ± 0.14	5	1.4			3.5		4.9
sertindole	−5.95 ± 0.06	5	−5.81 ± 0.07	5	−6.17 ± 0.03	5	1.4			1.7		2.3
olanzapine	−5.59 ± 0.05	5	−5.47 ± 0.06	5	−5.86 ± 0.02	5	1.3			1.9		2.5
paliperidone	−7.12 ± 0.04	5	−7.26 ± 0.05	5	−7.84 ± 0.03	5		1.4		5.2		3.8
risperidone	−7.30 ± 0.09	5	−7.47 ± 0.08	5	−8.04 ± 0.03	5		1.5		5.5		3.7
ziprasidone	−6.36 ± 0.11	5	−6.59 ± 0.08	5	−6.77 ± 0.08	5		1.7		2.6		1.5
clozapine	−5.86 ± 0.08^app^	5	−6.20 ± 0.05	5	−6.87 ± 0.08	5		2.2		10.2		4.7
lurasidone	−6.67 ± 0.05	5	−7.36 ± 0.06	5	−7.34 ± 0.03	5		4.9		4.7	1.0	
quetiapine	−5.81 ± 0.08	5	−6.72 ± 0.08	5	−6.66 ± 0.03	5		8.1		7.1	1.1	

Note

^app^ = apparent affinity. The maximum concentration of competing ligand inhibited most but not all of specific binding. An IC_50_ was determined by extrapolating the curve assuming that all specific binding would be inhibited if a higher concentration of competing ligand were possible.

^ep^ = early plateau, the competing ligand did not fully inhibit specific binding and the inhibition curve reached a plateau of maximal inhibition of binding. The specific binding inhibited by pimozide was 79.1 ± 6.0% at α2A.

### 3.3 Affinity and selectivity of ligands at β1 and β2‐adrenoceptors

Given that drug interactions at α1, α2, β1, and β2‐adrenoceptors affect blood pressure control, and that the affinity of these ligand has been assessed in comparative assays in α1 and α2 receptors, the affinity of ligands was also evaluated in CHO cells stably expressing the human β1 or β2‐adrenoceptor using ^3^H‐CGP12177 whole cell binding (Figure 3; Table 5).

**TABLE 5:** Log K_D_ values of ligands binding to the human β1 and β2‐adrenoceptors as measured by ^3^H‐CGP12177 whole cell binding. Values represent mean ±s.e.mean of n separate experiments. Ligands are arranged by class and presented in the same order as those in Tables 2, 3, and 4 for ease of comparison. Supplementary Table 1 has these ligands, alongside the α2‐data, presented in alphabetical order.

Ligand	Affinity measurements			
	Log K_D_ β1	n	Log K_D_ β2	n
α‐antagonists				
BRL44408	No binding to −3	5	No binding to −3	5
benoxathian	−4.55 ± 0.03^app^	5	−5.08 ± 0.06	5
tamsulosin	−6.26 ± 0.06	5	−6.08 ± 0.05	5
alfuzosin	No binding	5	−4.18 ± 0.09^app^	5
2‐MPMDQ	IC_50_>−5	6	IC_50_>−5	6
yohimbine	No binding to −4	5	No binding to −4	5
idazoxan	IC_50_>−3	5	IC_50_>−3	5
WB4104	IC_50_>−4	5	IC_50_>−4	5
A80426	−6.03 ± 0.05	6	−5.88 ± 0.04	6
eforaxan	no binding to −3	5	no binding to −3	5
2‐PMDQ	No binding −4	5	IC_50_>−4	5
atipamezole	No binding to −4.5	5	No binding to −4.5	5
RX821002	−4.55 ± 0.05	5	−3.95 ± 0.11^app^	5
sunepitron	IC_50_>−3	5	IC_50_>−3	5
doxazosin	−4.72 ± 0.06^app^	5	−5.57 ± 0.01	6
MK−912	IC_50_>−4	6	IC_50_>−4	6
phentolamine	IC_50_>−4	6	IC_50_>−4	6
RS17053	−5.44 ± 0.04	6	−6.42 ± 0.06	6
RS100329	IC_50_>−3	5	−4.77 ± 0.07	5
lisuride	−6.03 ± 0.06	5	−7.48 ± 0.04	5
BMY7378	IC_50_>−4	9	IC_50_>−4	9
RS79948	−3.84 ± 0.05	5	IC_50_>−3	5
carvedilol	−9.20 ± 0.05	8	−9.98 ± 0.06	8
JP1302	IC_50_>−4	5	−5.58 ± 0.08	5
SKF86466	−5.92 ± 0.08	6	−6.60 ± 0.07	6
3‐MPPI	No binding to −4	5	IC_50_>−4	5
PF3774076	No binding to −4	5	No binding to −4	5
Rec15‐2615	IC_50_>−4	5	IC_50_>−4	5
AH11110A	−6.23 ± 0.07	6	−6.36 ± 0.07	6
silodosin	IC_50_>−5	6	−7.52 ± 0.10	6
5‐methyl‐urapidil	−6.12 ± 0.04	5	−5.00 ± 0.07	5
SNAP5089	IC_50_>−5	5	IC_50_>−5	5
anisodamine	no binding to −3	9	no binding to −3	9
2‐niguldipine	IC_50_>−4	5	IC_50_>−4	5
naftapidil	−5.97 ± 0.07	6	−7.45 ± 0.06	6
labetolol	−7.97 ± 0.04	6	−8.21 ± 0.06	6
ifenprodil	IC_50_>−5	5	IC_50_>−5	5
domperidone	IC_50_>−4	5	IC_50_>−4	5
urapidil	−5.32 ± 0.06	5	−5.00 ± 0.02	5
HEAT	IC_50_ ~−4.5	5	IC_50_>−4	5
indoramin	−4.73 ± 0.10^app^	5	−5.27 ± 0.11^app^	5
cyclazosin	No binding to −4	6	−5.30 ± 0.04	6
imiloxan	IC_50_~−3	5	no binding to −3	5
dibenamine	−4.60 ± 0.06^app^	5	−4.94 ± 0.10^app^	5
promethazine	IC_50_>−4	10	IC_50_>−4	10
phenoxybenzamine	−4.36 ± 0.10^app^	5	−5.17 ± 0.13^app^	5
prazosin	No binding to −4	6	−5.10 ± 0.10^app^	5
terazosin	No binding to −4	4	No binding to −4	4
spiroxatrine	IC_50_>−4.5	5	IC_50_>−4.5	5
S32212	IC_50_>−5	5	IC_50_>−5	5
ARC239	IC_50_>−5	6	IC_50_>−5	5
β‐blockers				
S‐cyanopindolol	−10.39#		−11.09#	
bucindolol	−9.31#		−9.99#	
ICI118551	−6.61 ± 0.05	11	−9.41 ± 0.09	10
SDZ21009	−8.94#		−10.28#	
propranolol	−8.16*		−9.08*	
carazolol	−9.69#		−10.49#	
CGP12177	−9.21*		−9.39*	
CGP20712A	−8.87 ± 0.13	9	−5.74 ± 0.03	10
Tricyclic antidepressants				
clomipramine	IC_50_>−5	7	IC_50_>−5	7
protriptyline	IC_50_>−4	5	IC_50_>−4	5
norclomipramine	IC_50_>−4.5	10	IC_50_>−4.5	10
trimipramine	IC_50_>−4	5	IC_50_>−4	5
nortriptyline	−4.64 ± 0.13	5	−5.40 ± 0.08	5
desipramine	IC_50_>−4	5	−4.93 ± 0.03^app^	5
lofepramine	IC_50_>−4	4	IC_50_>−4	4
doxepin	IC_50_>−4	5	IC_50_>−4	5
dosulepin	IC_50_>−4	5	IC_50_>−4	5
imipramine	IC_50_>−4	5	IC_50_>−4	5
amitriptyline	IC_50_>−4	9	IC_50_>−4	9
Tetracyclic antidepressants				
mirtazepine	No binding to −4	5	No binding to −4	5
other noradrenaline and serotonin reuptake inhibitors				
duloxetine	IC_50_>−4.5		−6.07 ± 0.06	11
venlafaxime	−3.80 ± 0.11^app^	5	−4.13 ± 0.13^app^	5
Noradrenaline reuptake inhibitors				
reboxetine	IC_50_>−4	10	−5.26 ± 0.06	10
Selective serotonin reuptake inhibitors (SSRI)				
fluvoxamine	IC_50_>−4	10	IC_50_>−4	10
sertraline	IC_50_>−5	10	IC_50_>−5	10
fluoxetine	IC_50_>−4	10	IC_50_>−4	10
citalopram	No binding to −4	9	No binding to −4	9
paroxetine	IC_50_>−4.5	10	IC_50_>−4.5	10
Serotonin reuptake inhibitors				
vortioxetine	−6.37 ± 0.03	11	−6.75 ± 0.04	11
trazodone	IC_50_>−4	10	−5.14 ± 0.05	10
First‐generation antipsychotics				
sulpiride	IC_50_>−3	10	IC_50_>−3	10
haloperidol	IC_50_>−4	5	−4.94 ± 0.04^app^	5
flupenthixol	IC_50_>−5	10	IC_50_>−5	10
pimozide	IC_50_>−4	10	−5.75 ± 0.06	10
trifluoperazine	IC_50_>−5	10	IC_50_>−5	10
prochlorperazine	IC_50_>−5	10	IC_50_>−5	10
chlorpromazine	IC_50_>−5	5	IC_50_>−5	5
perphenazine	IC_50_>−5	10	IC_50_>−5	10
Second‐generation antipsychotics				
amisulpiride	No binding to −4	10	No binding to −4	10
aripirazole	−6.15 ± 0.04	6	−6.68 ± 0.08	6
sertindole	IC_50_>−5	5	IC_50_>−5	5
olanzapine	IC_50_>−3	4	−4.96 ± 0.05	4
paliperidone	IC_50_>−4.5	10	IC_50_>−4.5	10
risperidone	No binding to −4	5	IC_50_>−4	5
ziprasidone	No binding to −4	5	No binding to −4	5
clozapine	IC_50_>−5	5	IC_50_>−5	5
lurasidone	IC_50_>−5	10	IC_50_>−5	10
quetiapine	IC_50_>−4	10	IC_50_>−4	10

Note

#from^32^

*from^31^

Tables combining all ligands are presented in Supplementary Data. Supplementary Data Table 1 has the ligands arranged in alphabetical order (with suppliers and individual ligand codes, α2A, α2B, α2C, β1, and β2 affinity). Supplementary Data Table 2 has all ligands organised in order of α2A affinity (α2A, α2B, α2C affinities, and selectivities).

## 4 DISCUSSION

One aim of this study was to determine the selectivity of a range of ligands at the human α2‐adrenoceptors and this study confirmed previous comments that there are few α2‐subtype selective ligands.^11^, ^14^, ^15^, ^20^

### 4.1 Selectivity between α2A, α2B, and α2C‐adrenoceptors

Yohimbine and RX821002 were confirmed as high affinity antagonists at all three subtypes. Both compounds had a lower affinity at α2B‐adrenoceptors than at α2A or α2C, in keeping with some other studies (both in cell lines,^24^, ^29^ and in tissues.^7^, ^30^, ^39^, ^40^ Other compounds with high affinity at all 3 subtypes were: atipamezole^30^, ^39^ and RS79948^27^ and should thus be regarded as non‐selective α2‐ligands. Lisuride has a high affinity across many different receptor subtypes.^41^, ^42^

BRL44408 (65 nM at α2A) was the most α2A‐adrenoceptor selective ligand in keeping with^22^, ^24^, ^26^, ^43^ however although it was 60‐fold selective for α2A over α2B, BRL44408’s selectivity for α2A over α2C‐adrenoceptors was only 9‐fold. Although S32212 and ARC239 were 15‐to 21‐fold selective for the α2B over the α2A‐adrenoceptor, their α2B versus α2C is marginal (less than 5‐fold), in keeping with^21^, ^24^, ^28^, ^29^, ^43^, ^44^ and thus there are no α2B‐selective ligands. Within the α2‐adrenoceptors, JP1302 was the overall most α2C‐selective ligand with an α2C‐selectivity of 43 and 65 over α2A and α2B respectively, in keeping with^20^ however its affinity (120 nM at α2C) was a little lower than previously reported (16‐28 nM^20^). MK‐912 was the highest affinity ligand overall (0.15 nM at α2C) and also had some α2C‐selectivity (having 13 and 46‐fold higher α2C‐affinity than α2A or α2B respectively) again in keeping with previous studies.^24^, ^26^, ^27^, ^43^

Prazosin had higher affinity for α2C (257 nM) and α2B (676 nM) than α2A (4678 nM), and thus the pattern of affinity at these three subtypes was similar to some other studies of human receptors^24^, ^29^, ^30^ even if the absolute values have varied considerably (see Introduction for details).

### 4.2 Selectivity across α1, α2 and β‐adrenoceptors

Given that the affinity values determined in this study were using an identical technique to affinity values determined in the human α1 and β1 and β2‐adrenoceptors (the only difference was transfected receptor, radioligand and ligand used for non‐specific binding), a second aim of this study was to compare affinities between the human adrenoceptors (α2, β1, and β2 reported here, α1A, α1B, and α1D‐adrenoceptor subtypes from^33^ and β1, β2, and β3 from.^31^, ^32^). The findings of these studies are therefore discussed as a whole, in comparison with other literature findings.

**FIGURE 4:**
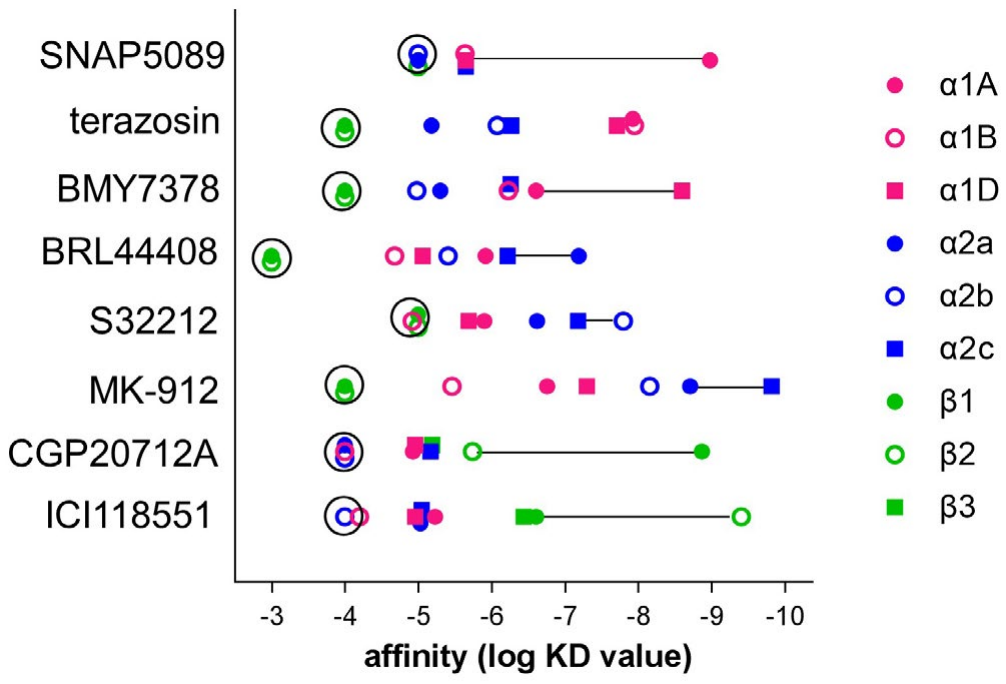
Plot of log K_D_ values showing the relative selectivity and affinity for the single most selective ligand at each receptor. Thus SNAP5089 is the most α1A‐selective ligand and the length of the line represents the selectivity for α1A over the next closest adrenoceptor affinity. Terazosin, although the “most” α1B‐selective ligand has no selectivity. The selectivity of the three most selective α2 ligands is considerably less than that for α1A, α1D, β1, or β2. Compounds within the black circles represent compounds where the log K_D_ is greater than the −3, −4, or −5 stated but included here to demonstrate attempts were made measurement. Data for α1‐adrenoceptors are from.^33^ β3 data are included for CGP20712A and ICI118551 from^31^

SNAP5089, silodosin and niguldipine are indeed highly α1A‐selective antagonists (>500 selectivity over α2 or β1 or β2‐adrenoceptors), and BMY7378 has ~100‐fold α1D‐selectivity. BRL44408 is the best α2A selective antagonist although its affinity for α2A is only a modest 9‐fold greater its α2C affinity. MK‐912 is the best α2C‐antagonist (0.15 nM α2C‐affntiy) although again its α2C selectivity is only modest (13‐fold greater than α2A). JP1302 (α2C affinity 120 nM) has an α1A‐adrenoceptor affinity of 617 nM, only 5‐fold less, so is not a truly α2C‐selective ligand. CGP20712A (β1) and ICI118551 (β2) are also highly selective antagonists with minimal α‐affinity. There are no truly α1B or α2B selective antagonists. Figure 4 shows the affinity (log K_D_ values) of the most selective ligand at each adrenoceptor subtype (i.e., BRL44408 for α2A, S32212 for α2B and MK‐912 for α2C) along with the single most selective antagonists at the other adrenoceptors and demonstrates that the α2‐adrenoceptors fall behind α1 and β with regards to availability of highly subtype‐selective ligands.

Silodosin (used for benign prostatic hyperplasia BPH) and naftopidil (used especially in Japan for BPH and ureteral stone expulsion,^45^) have significant β2‐adrenoceptor affinity (~30 nM). Silodosin is highly α1A‐selective (0.25 nM) giving a >100‐fold selectivity window compared to the other adrenoceptors. Naftopidil, however is not selective, with α1A and β2 affinities only 3‐fold apart and thus potentially increasing the risk of bronchospasm in those with asthma. Likewise, there is little evidence here to support SKF86466 being an α2‐selective antagonist.^46^, ^47^, ^48^ The affinity of SKF86466 for the β2‐adrenoceptor (250 nM) is similar to the highest α‐adrenoceptor affinity (407 nM at α2C). This may well be a species issue (see introduction) with previous studies being conducted in rodents,^46^, ^47^, ^48^ however others suggest a human α2A‐affinity of 13 nM.^23^

Labetolol and carvedilol are often usually referred to as dual α/β‐blockers (e.g.,.^49^). Labetolol (affinities of β2 6‐9 nM, β1 11‐23 nM, and α1A 47 nM) has very poor affinity at α1B, α1D, α2A, α2B, α2C, and β3‐adrenoceptors and thus reasonable affinity at only 1 out of 6 α‐adrenoceptors. A β/α1A‐antagonist would be a more accurate description. Likewise, carvedilol with affinities for β2 of 0.1–0.4 nM, β1 of 0.6–1.8 nM, and β3 of 5 nM also has highest α‐affinity for α1A (4 nM) over α1B or α1D (14 nM) or α2‐adrenoceptors (48‐490 nM), so with affinities up to 1000‐fold different across the 9 different adrenoceptors should not be considered a pan α/β‐blocker. The lack of affinity of other β‐blockers for the α‐adrenoceptors may also be expected.^50^

### 4.3 Antidepressants and antipsychotics

Given the considerable CNS expression of α2A and α2C‐adrenoceptors, and that many antidepressants and antipsychotics have high α1A‐affinity, a third aim of this study was to compare the affinity of antidepressants and antipsychotics across the adrenoceptors.

The antidepressants generally had poor α2‐adrenoceptor affinity, considerably lower affinity than that seen for the tricyclic antidepressant affinities at the α1A‐adrenoceptor. The antidepressant mirtazapine is a slight outsider with the highest α2‐affinity of the antidepressants studied here, and higher than α1A‐affinity. It has been associated with antinociceptive properties attributed to α2‐adrenoceptors in mice.^19^, ^51^ Mirtazepine (α2A‐affinity 158 nM) and α2C 110 nM), had similar affinity to the α2‐antagonist idazoxan and similar values to those obtained in human α2A receptors (79–126 nM) in,^51^ who also reported lower affinity at human α1 and unmeasurable affinity at human β1 or β2‐adrenoceptors. Of note,^51^ also reported similar values for mirtazapine for human and rat receptors, whereas^17^ suggest ~10‐fold higher α2‐affinity in what appears to be data gathered from mice.

Interestingly, many tricyclic antidepressants had a slight α2B‐selectivity, something not seen with most α‐ligands (Table 2), with the most potent (amitriptyline) having an α2B‐affinity (76 nM) only 10‐fold lower than that at the α1A‐adrenoceptor. Vortioxetine was the only antidepressant with any significant β‐adrenoceptor affinity and the only to have β‐adrenoceptor affinity greater than α‐adrenoceptor affinity (178 nM for the β2‐adrenoceptor).

α2C‐Adrenoceptor affinity has previously been suggested to have added benefits for the clinical actions of certain antipsychotics.^17^, ^52^ Here, first generation antipsychotics had lower affinity for the α2‐adrenoceptors than α1‐adrenoceptors, and had little selectivity for α2C over the other α2‐subtypes. For example, chlorpromazine had affinities of α2A 2239 nM, α2B 251 nM, α2C 1175 nM, whereas its α1A‐adrenoceptor affinity is 1 nM. There is however huge heterogeneity even between studies of human α2‐adrenoceptors. Chlorpromazine affinities range from α2A 78 nM, α2B 4.8 nM and α2C 41 nM (^3^H‐rauwloscine membrane binding from human receptors expressed in COS cells,^28^) α2A 396‐535 nM (^3^H‐yohimbine membrane binding using human colonic cancer cells and human platelets,^21^) α2A 600 nM, α2B 43 nM, and α2C 260 nM (^3^H‐RX821002 membrane binding for human receptors expressed in CHO cells,^29^) α2A 1008 nM, α2B 34 nM, and α2C 85 nM (^3^H‐RX821002 membrane binding to human receptors expressed in mouse cells,^30^) α2A 2245 nM (^3^H‐RX821002 membrane binding to human platelets,^23^) to α2A 4169 nM and α2C 1413 nM (antagonism of agonist responses living CHO cells expressing the human α2‐adrenoceptor^52^).

The second‐generation antipsychotics had a wide range of affinity for the α2‐adrenoceptors, with risperidone (9 nM, α2C) and paliperidone (14 nM α2C) having the highest affinity (in keeping with other human α2‐adrenoceptor studies^52^), to >1000 nM affinity for olazepine and amisulpiride. Even for risperidone and paliperidone, the α2C affinity is less potent than that seen at the α1A‐adrenoceptor and once again α2A vs α2C‐selectivity was very marginal. Clozepine, which has been particularly noted for α2C‐affinity^12^, ^13^, ^17^ had an α2C‐affinity of 135 nM, compared to its α1A‐affinity of 5.4 nM measured under identical conditions. This α2C affinity is similar to that measured in intact CHO cells expressing human receptors (54 nM,^52^ but poorer than that reported in membrane radioligand binding studies (6.5 nM^21^, ^29^).

### 4.4 Conclusion

This study, using identical methods to previous α1 and β‐adrenoceptor studies, allows comparison of ligand affinity, and thus selectivity, between the α and β‐adrenoceptor subtypes. Overall, there is huge variation in the literature for the affinity of α2 ligands (more so than for α1 or β), and for which species differences appear to play a significant role, but technique may also be important. Whilst selective antagonists exist for α1A, α1D, β1, and β2‐adrenoceptor, there are few selective α2‐adrenoceptor ligands and for those that do exist (BRL44408 for α1A and MK‐912 for α2C) only have small windows of selectivity. Antidepressants (with the exception of mirtazapine) and first‐generation antipsychotics have higher α1A than α2‐adrenoceptor affinity. Second‐generation antipsychotic varied widely in their α2‐adrenoceptor affinity, however, this study does not lend much support for an important role for an α2C‐selective action for certain antipsychotics. Clearly, however, even after a century of yohimbine use, there remains plenty of scope to develop selective α2‐antagonists.

## ETHICS STATEMENT

No animals, human tissue, human volunteers, or patients were used in this study.

## CONFLICTS OF INTEREST

JGB has been on the Scientific Advisory Board for CuraSen Therapeutics since 2019.

## AUTHOR CONTRIBUTION

JGB designed the research study. RGWP, JA, and JGB performed the research. JGB analyzed the data. JGB wrote the paper.

## Supporting information

Supplementary Material

## Data Availability

Further information and requests for data and reagents should be directed to and will be fulfilled by the corresponding author, Jillian Baker. Please contact jillian.baker@nottingham.ac.uk.
