# Supplementary material for: The affinity and selectivity of α‐adrenoceptor antagonists, antidepressants and antipsychotics for the human α2A, α2B, and α2C‐adrenoceptors and comparison with human α1 and β‐adrenoceptors
Source: Pharmacol Res Perspect. 2022 Feb 27;10(2):e00936. doi: 10.1002/prp2.936 (PMC8882856; doi:10.1002/prp2.936)
Supplement: Supplementary file 1 — Supplementary Material [file PRP2-10-e00936-s001.docx]

The affinity and selectivity of α-adrenoceptor antagonists, antidepressants and antipsychotics for the human α2A, α2B and α2C-adrenoceptors and comparison with human α1 and β-adrenoceptors.

## Richard G.W. Proudman, Juliana Akinaga, Jillian G. Baker

**Supplementary data**

Supplementary Data Table 1 – log K_D_ values arranged in alphabetical order of ligands

Supplementary Data Table 2 – log KD values arranged in order of α2A-adrenoceptor affinity

**Supplementary Data Table 1 – alphabetical order**

Alphabetical order for log K_D_ values of α-antagonists obtained from ^3^H-rauwolscine whole cell binding to the human α2A, α2B and α2C-adrenoceptors, and from ^3^H-CGP12177 whole cell binding to human β1 and β2-adrneoceptors stably expressed in CHO cells. Ligand suppliers and their catalogue numbers are also given. Values represent mean ± s.e.mean of n separate experiments.

|  |  |  | ^3^H-rauwolscine whole cell binding | | | | | |  | ^3^H-CGP12177 whole cell binding | | | | |
| --- | --- | --- | --- | --- | --- | --- | --- | --- | --- | --- | --- | --- | --- | --- |
| ligand | supplier | Catalogue  number | Log K_D_ α2A | n | Log K_D_ α2B | n | Log K_D_ α2C | n |  | Log K_D_ β1 | n |  | Log K_D_ β2 | n |
| A80426 | Tocris | 2341 | -7.24 ± 0.08 | 6 | -6.52 ± 0.06 | 6 | -7.46 ± 0.07 | 6 |  | -6.03 ± 0.05 | 6 |  | -5.88 ± 0.04 | 6 |
| AH11110A | Sigma | A3477 | -4.70 ± 0.04^app^ | 5 | IC_50_>-4 | 5 | -4.86 ± 0.03^app^ | 5 |  | -6.23 ± 0.07 | 6 |  | -6.36 ± 0.07 | 6 |
| Alfuzosin | Sellakchem | S1409 | -5.56 ± 0.04 | 5 | -4.62 ± 0.05 | 5 | -6.14 ± 0.04 | 5 |  | No binding to -4 | 5 |  | -4.18 ± 0.09^app^ | 5 |
| amisulpiride | Sellakchem | S1280 | -5.11 ± 0.09^app^ | 5 | -4.69 ± 0.13^app^ | 5 | -5.57 ± 0.07 | 5 |  | No binding to -4 | 10 |  | No binding to -4 | 10 |
| amitriptyline | Sigma | A8404 | -5.86 ± 0.05^app^ | 5 | -7.12 ± 0.05 | 5 | -6.67 ± 0.09 | 5 |  | IC_50_>-4 | 9 |  | IC_50_>-4 | 9 |
| anisodamine | Sigma | SML0252 | IC_50_>-3 | 5 | IC_50_>-3 | 5 | -3.56 ± 0.07^app^ | 5 |  | no binding to -3 | 9 |  | no binding to -3 | 9 |
| ARC 239 | Sigma | A5736 | -5.99 ± 0.06 | 5 | -7.32 ± 0.14 | 6 | -7.25 ± 0.14 | 5 |  | IC_50_>-5 | 6 |  | IC_50_>-5 | 5 |
| aripirazole | Tocris | 5584 | -6.68 ± 0.08 | 5 | -6.54 ± 0.08 | 6 | -7.23 ± 0.14 | 5 |  | -6.15 ± 0.04 | 6 |  | -6.68 ± 0.08 | 6 |
| atipamezole | Sigma | A9611 | -8.50 ± 0.08 | 5 | -7.85 ± 0.04 | 5 | -8.48 ± 0.09 | 5 |  | No binding to -4.5 | 5 |  | No binding to -4.5 | 5 |
| benoxathian | Sigma | B016 | -7.17 ± 0.02 | 5 | -5.96 ± 0.06 | 5 | -7.75 ± 0.03 | 5 |  | -4.55 ± 0.03^app^ | 5 |  | -5.08 ± 0.06 | 5 |
| BRL44408 | Sigma | B4559 | -7.19 ± 0.04 | 7 | -5.41 ± 0.04 | 7 | -6.22 ± 0.07 | 7 |  | No binding to -3 | 5 |  | No binding to -3 | 5 |
| bucindolol | Tocris | 2658 | -5.81 ± 0.05 | 5 | -5.63 ± 0.06 | 5 | -5.95 ± 0.04 | 5 |  | -9.31# |  |  | -9.99# |  |
| BMY7378 | Sellakchem | S2691 | -5.30 ± 0.03 | 5 | -4.98 ± 0.09^app^ | 5 | -6.26 ± 0.01 | 5 |  | IC_50_>-4 | 9 |  | IC_50_>-4 | 9 |
| carazolol | Sigma | 53787 | -4.66 ± 0.06^app^ | 6 | IC_50_>-4 | 6 | -4.66 ± 0.05^app^ | 6 |  | -9.69# |  |  | -10.49# |  |
| carvedilol | Tocris | 2685 | -6.54 ± 0.02 | 5 | -6.31 ± 0.02 | 5 | -7.32 ± 0.05 | 5 |  | -9.20 ± 0.05 | 8 |  | -9.98 ± 0.06 | 8 |
| CGP12177 | Sigma | C125 | IC_50_>-3 | 5 | No bind to -3 | 5 | IC_50_>-3 | 5 |  | -9.21* |  |  | -9.39* |  |
| CGP20712A | Tocris | 1024 | IC_50_>-4 | 5 | IC_50_>-4 | 5 | -5.17 ± 0.03 | 5 |  | -8.87 ± 0.13 | 9 |  | -5.74 ± 0.03 | 10 |
| chlorpromazine | Sigma | C8138 | -5.65 ± 0.13^app^ | 6 | -6.60 ± 0.12 | 6 | -5.93 ± 0.11 | 6 |  | IC_50_>-5 | 5 |  | IC_50_>-5 | 5 |
| citalopram | Tocris | 1427 | IC_50_>-4 | 5 | IC_50_>-4 | 5 | IC_50_>-4 | 5 |  | No binding to -4 | 9 |  | No binding to -4 | 9 |
| clomipramine | Sigma | C7291 | -5.71 ± 0.07^app^ | 5 | -6.10 ± 0.13 | 5 | -5.80 ± 0.02^app^ | 5 |  | IC_50_>-5 | 7 |  | IC_50_>-5 | 7 |
| clozapine | Sigma | 16305 | -5.86 ± 0.08^app^ | 5 | -6.20 ± 0.05 | 5 | -6.87 ± 0.08 | 5 |  | IC_50_>-5 | 5 |  | IC_50_>-5 | 5 |
| S-cyanopindolol | Tocris | 0993 | -5.56 ± 0.10 | 5 | -4.82 ± 0.10^app^ | 5 | -6.15 ± 0.07 | 5 |  | -10.39# |  |  | -11.09# |  |
| cyclazosin | Sigma | C247 | -5.00 ± 0.03 | 5 | -5.35 ± 0.13 | 5 | -6.18 ± 0.02 | 5 |  | No binding to -4 | 6 |  | -5.30 ± 0.04 | 6 |
| desipramine | Sigma | D3900 | -5.04 ± 0.06 | 5 | -5.78 ± 0.04 | 5 | -5.52 ± 0.03 | 5 |  | IC_50_>-4 | 5 |  | -4.93 ± 0.03^app^ | 5 |
| dibenamine | Sigma | 291366 | -5.80 ± 0.06 | 10 | -6.43 ± 0.06  -4.64 ± 0.07  60.9 ± 3.4% site 1 | 10 | -6.18 ± 0.05 | 10 |  | -4.60 ± 0.06^app^ | 5 |  | -4.94 ± 0.10^app^ | 5 |
| domperidone | Sigma | D122 | -5.09 ± 0.06^app^ | 6 | -5.29 ± 0.07 | 6 | -5.78 ± 0.08 | 6 |  | IC_50_>-4 | 5 |  | IC_50_>-4 | 5 |
| dosulepin | EPRS | D2962000 | -5.16 ± 0.06 | 5 | -6.20 ± 0.06 | 5 | -5.63 ± 0.11 | 5 |  | IC_50_>-4 | 5 |  | IC_50_>-4 | 5 |
| doxepin | Tocris | 0508 | -5.69 ± 0.12 | 5 | -6.67 ± 0.05 | 5 | -6.04 ± 0.07 | 5 |  | IC_50_>-4 | 5 |  | IC_50_>-4 | 5 |
| doxazosin | Sigma | D985 | -5.35 ± 0.04 | 6 | -4.74 ± 0.07^app^ | 6 | -6.24 ± 0.02 | 6 |  | -4.72 ± 0.06^app^ | 5 |  | -5.57 ± 0.01 | 6 |
| duloxetine | sigma | PHR1865 | -5.43 ± 0.06 | 5 | -5.31 ± 0.09 | 5 | -5.67 ± 0.06 | 5 |  | IC_50_>-4.5 | 5 |  | -6.07 ± 0.06 | 11 |
| eforaxan | Tocris | 0792 | -7.58 ± 0.05 | 5 | -6.88 ± 0.07 | 5 | -7.44 ± 0.04 | 5 |  | no binding to -3 | 5 |  | no binding to -3 | 5 |
| fluoxetine | Sellakchem | S1333 | -4.70 ± 0.10^app^ | 5 | -4.99 ± 0.03 | 5 | -4.79 ± 0.07^app^ | 5 |  | IC_50_>-4 | 10 |  | IC_50_>-4 | 10 |
| flupenthixol | Tocris | 4057 | -6.10 ± 0.12 | 5 | -6.28 ± 0.13 | 5 | -6.88 ± 0.14 | 5 |  | IC_50_>-5 | 10 |  | IC_50_>-5 | 10 |
| fluvoxamine | Sellakchem | S1336 | -4.81 ± 0.04^app^ | 6 | -4.37 ± 0.08app | 5 | -4.82 ± 0.07^app^ | 6 |  | IC_50_>-4 | 10 |  | IC_50_>-4 | 10 |
| Haloperidol | Sigma | H1512 | -5.38 ± 0.06 | 5 | -5.53 ± 0.10 | 5 | -5.77 ± 0.05 | 5 |  | IC_50_>-4 | 5 |  | -4.94 ± 0.04^app^ | 5 |
| HEAT | Tocris | 0535 | -7.45 ± 0.04 | 5 | -7.72 ± 0.11 | 5 | -8.05 ± 0.19 | 5 |  | IC_50_ ~-4.5 | 5 |  | IC_50_>-4 | 5 |
| ICI 118551 | Sigma | I127 | -5.03 ± 0.03 | 5 | IC_50_>-4 | 5 | -5.05 ± 0.04 | 5 |  | -6.61 ± 0.05 | 11 |  | -9.41 ± 0.09 | 10 |
| idazoxan | Sigma | I6138 | -7.17 ± 0.04 | 5 | -6.39 ± 0.05 | 5 | -7.16 ± 0.03 | 5 |  | IC_50_>-3 | 5 |  | IC_50_>-3 | 5 |
| ifenprodil | Sellakchem | S4091 | -6.01 ± 0.05 | 5 | -6.14 ± 0.06 | 5 | -6.80 ± 0.05 | 5 |  | IC_50_>-5 | 5 |  | IC_50_>-5 | 5 |
| imipramine | Sigma | I0899 | -5.25 ± 0.04 | 5 | -6.36 ± 0.08 | 5 | -5.89 ± 0.03 | 5 |  | IC_50_>-4 | 5 |  | IC_50_>-4 | 5 |
| imiloxan | Sigma | I9531 | -5.88 ± 0.03 | 6 | -6.48 ± 0.05 | 6 | -6.27 ± 0.03 | 6 |  | IC_50_~-3 | 5 |  | no binding -3 | 5 |
| indoramin | Sigma | I2909 | -5.13 ± 0.03^app^ | 6 | -5.46 ± 0.05 | 6 | -5.80 ± 0.05 | 6 |  | -4.73 ± 0.10^app^ | 5 |  | -5.27 ± 0.11^app^ | 5 |
| JP1302 | Tocris | 2666 | -5.29 ± 0.04 | 5 | -5.11 ± 0.05 | 5 | -6.92 ± 0.13 | 5 |  | IC_50_>-4 | 5 |  | -5.58 ± 0.08 | 5 |
| labetolol | Sigma | L1011 | -4.62 ± 0.07^app^ | 5 | -4.71 ± 0.08^app^ | 5 | -5.27 ± 0.04 | 5 |  | -7.97 ± 0.04 | 6 |  | -8.21 ± 0.06 | 6 |
| lisuride | Tocris | 4052 | -8.99 ± 0.05 | 5 | -8.52 ± 0.05 | 5 | -9.27 ± 0.05 | 5 |  | -6.03 ± 0.06 | 5 |  | -7.48 ± 0.04 | 5 |
| lurasidone | Sellakchem | S3044 | -6.67 ± 0.05 | 5 | -7.36 ± 0.06 | 5 | -7.34 ± 0.03 | 5 |  | IC_50_>-5 | 10 |  | IC_50_>-5 | 10 |
| lofepramine | Tocris | 2545 | -4.86 ± 0.04^app^ | 5 | -5.60 ± 0.08 | 5 | -5.28 ± 0.06 | 5 |  | IC_50_>-4 | 5 |  | IC_50_>-4 | 5 |
| 5-methyl-urapidil | Sigma | H101 | -5.18 ± 0.05 | 5 | -5.17 ± 0.05 | 5 | -5.81 ± 0.07 | 5 |  | -6.12 ± 0.04 | 5 |  | -5.00 ± 0.07 | 5 |
| MK-912 | Sigma | M7065 | -8.71 ± 0.05 | 8 | -8.16 ± 0.10 | 8 | -9.82 ± 0.11 | 9 |  | IC_50_>-4 | 6 |  | IC_50_>-4 | 6 |
| mirtazepine | Tocris | 2018 | -6.80 ± 0.05 | 5 | -6.09 ± 0.06 | 5 | -6.96 ± 0.03 | 5 |  | No binding to -4 | 5 |  | No binding to -4 | 5 |
| 2-MPMDQ | Tocris | 0661 | -6.79 ± 0.04 | 5 | -5.94 ± 0.09 | 5 | -7.50 ± 0.02 | 5 |  | IC_50_>-5 | 6 |  | IC_50_>-5 | 6 |
| 3-MPPI | Tocris | 0581 | -6.67 ± 0.05^ep^ | 5 | IC_50_>-4 | 5 | -7.01 ± 0.03^ep^ | 5 |  | No binding to -4 | 5 |  | IC_50_>-4 | 5 |
| naftapidil | Tocris | 0597 | -6.55 ± 0.09 | 5 | -6.60 ± 0.07 | 5 | -7.17 ± 0.08 | 5 |  | -5.97 ± 0.07 | 6 |  | -7.45 ± 0.06 | 6 |
| 2-niguldipine | Tocris | 1123 | IC_50_>-5 | 5 | -5.48 ± 0.11 | 5 | -6.07 ± 0.11 | 5 |  | IC_50_>-4 | 5 |  | IC_50_>-4 | 5 |
| Norclomipramine | sigma | N1280 | -5.29 ± 0.09^app^ | 6 | -5.74 ± 0.04^app^ | 6 | -5.80 ± 0.07^app^ | 6 |  | IC_50_>-4.5 | 10 |  | IC_50_>-4.5 | 10 |
| nortriptyline | Sigma | N7261 | -5.65 ± 0.05 | 5 | -6.38 ± 0.02 | 5 | -6.19 ± 0.08 | 5 |  | -4.64 ± 0.13 | 5 |  | -5.40 ± 0.08 | 5 |
| olanzapine | Sigma | O1141 | -5.59 ± 0.05 | 5 | -5.47 ± 0.06 | 5 | -5.86 ± 0.02 | 5 |  | IC_50_>-3 | 5 |  | IC_50~_>-4 | 5 |
| 2-PMDQ | Tocris | 0627 | -6.83 ± 0.05 | 5 | -6.14 ± 0.08 | 5 | -7.07 ± 0.02 | 5 |  | No binding to -4 | 5 |  | IC_50_>-4 | 5 |
| paliperidone | Sellakchem | S1724 | -7.12 ± 0.04 | 5 | -7.26 ± 0.05 | 5 | -7.84 ± 0.03 | 5 |  | IC_50_>-4.5 | 10 |  | IC_50_>-4.5 | 10 |
| paroxetine | Sellakcham | S3005 | IC_50_>-5 | 5 | IC_50_>-5 | 5 | IC_50_>-5 | 5 |  | IC_50_>-4.5 | 10 |  | IC_50_>-4.5 | 10 |
| perphenazine | Sellakchem | S4731 | -6.00 ± 0.06 | 6 | -7.16 ± 0.05 | 6 | -6.83 ± 0.04 | 5 |  | IC_50_>-5 | 10 |  | IC_50_>-5 | 10 |
| PF3774076 |  |  | -5.59 ± 0.04 | 6 | IC_50_>-4 | 6 | -5.29 ± 0.09 | 6 |  | No binding to -4 | 5 |  | No binding to -4 | 5 |
| phenoxybenzamine | Sigma | B019 | -5.72 ± 0.10 | 10 | -6.44 ± 0.11  -4.89 ± 0.08  51.4 ± 3.3% site 1 | 10 | -6.41 ± 0.11  -4.71 ± 0.13  74.1 ± 4.1% site 1 | 10 |  | -4.36 ± 0.10^app^ | 5 |  | -5.17 ± 0.13^app^ | 5 |
| phentolamine | Sigma | P7547 | -7.26 ± 0.03 | 5 | -6.69 ± 0.05 | 5 | -6.92 ± 0.04 | 5 |  | IC_50_>-4 | 6 |  | IC_50_>-4 | 6 |
| pimozide | Tocris | 0937 | -5.76 ± 0.12^ep^ | 5 | -6.30 ± 0.10 | 5 | -6.84 ± 0.05 | 5 |  | IC_50_>-4 | 10 |  | -5.75 ± 0.06 | 10 |
| prazosin | Tocris | 0623 | -5.33 ± 0.05 | 6 | -6.17 ± 0.05 | 6 | -6.59 ± 0.04 | 6 |  | No binding to -4 | 6 |  | -5.10 ± 0.10^app^ | 5 |
| prochlorperazine | Sigma | P9178 | -5.78 ± 0.02^app^ | 6 | -6.46 ± 0.11 | 6 | -6.31 ± 0.09 | 6 |  | IC_50_>-5 | 10 |  | IC_50_>-5 | 10 |
| promethazine | Sigma | P4651 | -5.58 ± 0.07 | 5 | -6.25 ± 0.06 | 5 | -5.54 ± 0.05 | 5 |  | IC_50_>-4 | 10 |  | IC_50_>-4 | 10 |
| propranolol | Sigma | P0884 | -4.85 ± 0.02 | 5 | IC_50_>-4 | 5 | -4.71 ± 0.06 | 5 |  | -8.16* |  |  | -9.08* |  |
| protriptyline | Sigma | P8813 | -5.00 ± 0.05 | 5 | -5.39 ± 0.13 | 5 | -5.26 ± 0.07 | 5 |  | IC_50_>-4 | 5 |  | IC_50_>-4 | 5 |
| quetiapine | Sellakchem | S1763 | -5.81 ± 0.08 | 5 | -6.72 ± 0.08 | 5 | -6.66 ± 0.03 | 5 |  | IC_50_>-4 | 10 |  | IC_50_>-4 | 10 |
| Rec15-2615 | Tocris | 3284 | -5.53 ± 0.12^app^ | 6 | IC50>-4.5 | 6 | -6.56 ± 0.13 | 6 |  | IC_50_>-4 | 5 |  | IC_50_>-4 | 5 |
| reboxetine | Sellakchem | S3199 | IC_50_>-4 | 5 | IC_50_>-4 | 5 | -4.56 ± 0.07^app^ | 4 |  | IC_50_>-4 | 10 |  | -5.26 ± 0.06 | 10 |
| risperidone | Sigma | R3030 | -7.30 ± 0.09 | 5 | -7.47 ± 0.08 | 5 | -8.04 ± 0.03 | 5 |  | No binding to -4 | 5 |  | IC_50_>-4 | 5 |
| RS100329 | Tocris | 1352 | -7.00 ± 0.03 | 5 | -6.47 ± 0.04 | 5 | -7.82 ± 0.03 | 5 |  | IC_50_>-3 | 5 |  | -4.77 ± 0.07 | 5 |
| RS17053 | Tocris | 0985 | -6.20 ± 0.11 | 5 | -5.65 ± 0.07 | 5 | -6.35 ± 0.08 | 5 |  | -5.44 ± 0.04 | 6 |  | -6.42 ± 0.06 | 6 |
| RS79948 | Tocris | 0987 | -8.93 ± 0.03 | 5 | -8.57 ± 0.03 | 5 | -9.36 ± 0.04 | 5 |  | -3.84 ± 0.05 | 5 |  | IC_50_>-3 | 5 |
| RX 821002 | Sigma | R9525 | -8.10 ± 0.07 | 5 | -7.45 ± 0.06 | 5 | -8.14 ± 0.02 | 5 |  | -4.55 ± 0.05 | 5 |  | -3.95 ± 0.11^app^ | 5 |
| S32212 | Tocris | 4508 | -6.62 ± 0.13 | 8 | -7.80 ± 0.10 | 8 | -7.18 ± 0.10 | 8 |  | IC_50_>-5 | 5 |  | IC_50_>-5 | 5 |
| SDZ21009 | Tocris | 1516 | -4.86 ± 0.07^app^ | 6 | IC_50_>-4 | 6 | IC_50_>-4.5 | 6 |  | -8.94# |  |  | -10.28# |  |
| sertindole | Sigma | S8072 | -5.95 ± 0.06 | 5 | -5.81 ± 0.07 | 5 | -6.17 ± 0.03 | 5 |  | IC_50_>-5 | 5 |  | IC_50_>-5 | 5 |
| sertraline | Sellakchem | S4053 | -5.67 ± 0.07^app^ | 6 | -5.62 ± 0.11^app^ | 6 | -5.64 ± 0.05^app^ | 6 |  | IC_50_>-5 | 10 |  | IC_50_>-5 | 10 |
| silodosin | Sellakchem | S1613 | -5.49 ± 0.06^app^ | 6 | IC_50_>-5 | 6 | -6.12 ± 0.06^app^ | 6 |  | IC_50_>-5 | 6 |  | -7.52 ± 0.10 | 6 |
| SKF86466 | Tocris | 3866 | -6.29 ± 0.05 | 5 | -6.17 ± 0.047 | 5 | -6.39 ± 0.04 | 5 |  | -5.92 ± 0.08 | 6 |  | -6.60 ± 0.07 | 6 |
| SNAP5089 | Tocris | 2398 | IC_50_>-5 | 5 | IC_50_>-5 | 5 | -5.65 ± 0.06 | 5 |  | IC_50_>-5 | 5 |  | IC_50_>-5 | 5 |
| spiroxatrine | Tocris | 0631 | -6.97 ± 0.03 | 6 | -7.87 ± 0.07 | 6 | -8.74 ± 0.04 | 6 |  | IC_50_>-4.5 | 5 |  | IC_50_>-4.5 | 5 |
| sulpiride | Sigma | S8010 | -4.50 ± 0.02 | 5 | -4.37 ± 0.06 | 5 | -4.67 ± 0.07 | 5 |  | IC_50_>-3 | 10 |  | IC_50_>-3 | 10 |
| sunepitron | Sigma | PZ0279 | -7.28 ± 0.04 | 6 | -6.65 ± 0.08 | 6 | -8.11 ± 0.04 | 6 |  | IC_50_>-3 | 5 |  | IC_50_>-3 | 5 |
| tamsulosin | Sigma | T1330 | -6.33 ± 0.04 | 5 | -5.31 ± 0.04 | 5 | -6.41 ± 0.03 | 5 |  | -6.26 ± 0.06 | 5 |  | -6.08 ± 0.05 | 5 |
| terazosin | Tocris | 1506 | -5.18 ± 0.03 | 5 | -6.08 ± 0.05 | 5 | -6.27 ± 0.08 | 5 |  | No binding to -4 | 5 |  | No binding to -4 | 5 |
| trazodone | Sigma | T6154 | -6.17 ± 0.08 | 5 | -5.96 ± 0.07 | 5 | -6.69 ± 0.04 | 5 |  | IC_50_>-4 | 10 |  | -5.14 ± 0.05 | 10 |
| trifluoperazine | Sigma | T8516 | -5.60 ± 0.05 | 5 | -6.22 ± 0.12 | 5 | -6.20 ± 0.06 | 5 |  | IC_50_>-5 | 10 |  | IC_50_>-5 | 10 |
| trimipramine | Sigma | T3146 | -5.67 ± 0.03 | 5 | -6.22 ± 0.05 | 5 | -6.37 ± 0.03 | 5 |  | IC_50_>-4 | 5 |  | IC_50_>-4 | 5 |
| urapidil | Tocris | 1772 | -5.49 ± 0.05 | 5 | -5.78 ± 0.08 | 5 | -6.34 ± 0.05 | 5 |  | -5.32 ± 0.06 | 5 |  | -5.00 ± 0.02 | 5 |
| venlafaxime | Sellakchem | S1441 | -3.46 ± 0.03^app^ | 5 | IC_50_>-3 | 5 | -3.74 ± 0.11^app^ | 5 |  | -3.80 ± 0.11app | 5 |  | -4.13 ± 0.13app | 5 |
| vortioxetine | Sellakchem | S8021 | -5.63 ± 0.06^app^ | 5 | -5.32 ± 0.04^app^ | 6 | -5.84 ± 0.05 | 6 |  | -6.37 ± 0.03 | 11 |  | -6.75 ± 0.04 | 11 |
| WB4104 | Tocris | 0946 | -7.55 ± 0.05 | 6 | -6.77 ± 0.05 | 6 | -8.17 ± 0.05 | 6 |  | IC_50_>-4 | 5 |  | IC_50_>-4 | 5 |
| yohimbine | Sigma | Y3125 | -8.48 ± 0.07 | 5 | -7.66 ± 0.10 | 5 | -8.52 ± 0.05 | 5 |  | No binding to -4 | 5 |  | No binding to -4 | 5 |
| ziprasidone | Sellakchem | S1444 | -6.36 ± 0.11 | 5 | -6.59 ± 0.08 | 5 | -6.77 ± 0.08 | 5 |  | No binding to -4 | 5 |  | No binding to -4 | 5 |

^app^ = apparent affinity. The maximum concentration of competing ligand inhibited most but not all of specific binding. An IC_50_ was determined by extrapolating the curve assuming that all specific binding would be inhibited if a higher concentration of competing ligand were possible. Thus an apparent K_D_ was calculated.

^ep^ = early plateau, the competing ligand did not fully inhibit specific binding and the inhibition curve reached a plateau of maximal inhibition of binding. The specific binding inhibited by 3-MPPI was 75.6 ± 0.9% at α2A and 87.1 ± 1.5% at α2C and for pimozide was 79.1 ± 6.0% at α2A.

#from [29]

*from [28]

**Supplementary Data Table 2 – in order of α2A-adrenoceptor affinity**

Affinity (log K_D_ values) of α-antagonists obtained from ^3^H-rauwolscine whole cell binding to the human α2A, α2B and α2C-adrenoceptors stably expressed in CHO cells. Ligands are arranged in order of α2A-adrenoceptor affinity. Values represent mean ± s.e.mean of n separate experiments. Selectivity ratios are also given where a ratio of 1 demonstrates no selectivity for a given receptor subtype over another.

|  | Log K_D_ values determined from ^3^H-rauwolscine whole cell binding | | | | | |  | Selectivity ratios | | | | | |
| --- | --- | --- | --- | --- | --- | --- | --- | --- | --- | --- | --- | --- | --- |
| ligand | Log K_D_ α2A | n | Log K_D_ α2B | n | Log K_D_ α2C | n |  | α2A vs α2B | | α2A vs α2C | | α2B vs α2C | |
| lisuride | -8.99 ± 0.05 | 5 | -8.52 ± 0.05 | 5 | -9.27 ± 0.05 | 5 |  | 3.0 |  |  | 1.9 |  | 5.6 |
| RS79948 | -8.93 ± 0.03 | 5 | -8.57 ± 0.03 | 5 | -9.36 ± 0.04 | 5 |  | 2.3 |  |  | 2.7 |  | 6.2 |
| MK-912 | -8.71 ± 0.05 | 8 | -8.16 ± 0.10 | 7 | -9.82 ± 0.11 | 9 |  | 3.6 |  |  | 12.9 |  | 45.7 |
| atipamezole | -8.50 ± 0.08 | 5 | -7.85 ± 0.04 | 5 | -8.48 ± 0.09 | 5 |  | 4.5 |  | 1.0 | |  | 4.3 |
| yohimbine | -8.48 ± 0.07 | 5 | -7.66 ± 0.10 | 5 | -8.52 ± 0.05 | 5 |  | 6.6 |  |  | 1.1 |  | 7.2 |
| RX 821002 | -8.10 ± 0.07 | 5 | -7.45 ± 0.06 | 5 | -8.14 ± 0.02 | 5 |  | 4.5 |  |  | 1.1 |  | 4.9 |
| eforaxan | -7.58 ± 0.05 | 5 | -6.88 ± 0.07 | 5 | -7.44 ± 0.04 | 5 |  | 5.0 |  | 1.4 |  |  | 3.6 |
| WB4104 | -7.55 ± 0.05 | 6 | -6.77 ± 0.05 | 6 | -8.17 ± 0.05 | 6 |  | 6.0 |  |  | 4.2 |  | 25.1 |
| HEAT | -7.45 ± 0.04 | 5 | -7.72 ± 0.11 | 5 | -8.05 ± 0.19 | 5 |  |  | 1.9 |  | 4.0 |  | 2.1 |
| risperidone | -7.30 ± 0.09 | 5 | -7.47 ± 0.08 | 5 | -8.04 ± 0.03 | 5 |  |  | 1.5 |  | 5.5 |  | 3.7 |
| sunepitron | -7.28 ± 0.04 | 6 | -6.65 ± 0.08 | 6 | -8.11 ± 0.04 | 6 |  | 4.3 |  |  | 6.8 |  | 28.8 |
| phentolamine | -7.26 ± 0.03 | 5 | -6.69 ± 0.05 | 5 | -6.92 ± 0.04 | 5 |  | 3.7 |  | 2.2 |  |  | 1.7 |
| A80426 | -7.24 ± 0.08 | 6 | -6.52 ± 0.06 | 6 | -7.46 ± 0.07 | 6 |  | 5.2 |  |  | 1.7 |  | 8.7 |
| benoxathian | -7.17 ± 0.02 | 5 | -5.96 ± 0.06 | 5 | -7.75 ± 0.03 | 5 |  | 16.2 |  |  | 3.8 |  | 61.7 |
| BRL44408 | -7.19 ± 0.04 | 7 | -5.41 ± 0.04 | 7 | -6.22 ± 0.07 | 7 |  | 60.3 |  | 9.3 |  |  | 6.5 |
| idazoxan | -7.17 ± 0.04 | 5 | -6.39 ± 0.05 | 5 | -7.16 ± 0.03 | 5 |  | 6.0 |  | 1.0 | |  | 5.9 |
| paliperidone | -7.12 ± 0.04 | 5 | -7.26 ± 0.05 | 5 | -7.84 ± 0.03 | 5 |  |  | 1.4 |  | 5.2 |  | 3.8 |
| RS100329 | -7.00 ± 0.03 | 5 | -6.47 ± 0.04 | 5 | -7.82 ± 0.03 | 5 |  | 3.4 |  |  | 6.6 |  | 22.4 |
| spiroxatrine | -6.97 ± 0.03 | 6 | -7.87 ± 0.07 | 6 | -8.74 ± 0.04 | 6 |  |  | 7.9 |  | 58.9 |  | 7.4 |
| 2-PMDQ | -6.83 ± 0.05 | 5 | -6.14 ± 0.08 | 5 | -7.07 ± 0.02 | 5 |  | 4.9 |  |  | 1.7 |  | 8.5 |
| mirtazepine | -6.80 ± 0.05 | 5 | -6.09 ± 0.06 | 5 | -6.96 ± 0.03 | 5 |  | 5.1 |  |  | 1.4 |  | 7.4 |
| 2-MPMDQ | -6.79 ± 0.04 | 5 | -5.94 ± 0.09 | 5 | -7.50 ± 0.02 | 5 |  | 7.1 |  |  | 5.1 |  | 36.3 |
| aripirazole | -6.68 ± 0.08 | 5 | -6.54 ± 0.05 | 6 | -7.23 ± 0.14 | 5 |  | 1.4 |  |  | 3.5 |  | 4.9 |
| lurasidone | -6.67 ± 0.05 | 5 | -7.36 ± 0.06 | 5 | -7.34 ± 0.03 | 5 |  |  | 4.9 |  | 4.7 | 1.0 | |
| 3-MPPI | -6.67 ± 0.05^ep^ | 5 | IC_50_>-4 | 5 | -7.01 ± 0.03^ep^ | 5 |  |  |  |  | 2.2 |  |  |
| naftapidil | -6.55 ± 0.09 | 5 | -6.60 ± 0.07 | 5 | -7.17 ± 0.08 | 5 |  |  | 1.1 |  | 4.2 |  | 3.7 |
| S32212 | -6.62 ± 0.13 | 8 | -7.80 ± 0.10 | 8 | -7.18 ± 0.10 | 8 |  |  | 15.1 |  | 3.6 | 4.2 |  |
| carvedilol | -6.54 ± 0.02 | 5 | -6.31 ± 0.02 | 5 | -7.32 ± 0.05 | 5 |  | 1.7 |  |  | 6.0 |  | 10.2 |
| ziprasidone | -6.36 ± 0.11 | 5 | -6.59 ± 0.08 | 5 | -6.77 ± 0.08 | 5 |  |  | 1.7 |  | 2.6 |  | 1.5 |
| tamsulosin | -6.33 ± 0.04 | 5 | -5.31 ± 0.04 | 5 | -6.41 ± 0.03 | 5 |  | 10.5 |  |  | 1.2 |  | 12.6 |
| SKF86466 | -6.29 ± 0.05 | 5 | -6.17 ± 0.047 | 5 | -6.39 ± 0.04 | 5 |  | 1.3 |  |  | 1.3 |  | 1.7 |
| RS17053 | -6.20 ± 0.11 | 5 | -5.65 ± 0.07 | 5 | -6.35 ± 0.08 | 5 |  | 3.5 |  |  | 1.4 |  | 5.0 |
| trazodone | -6.17 ± 0.08 | 5 | -5.96 ± 0.07 | 5 | -6.69 ± 0.04 | 5 |  | 1.6 |  |  | 3.3 |  | 5.4 |
| ifenprodil | -6.01 ± 0.05 | 5 | -6.14 ± 0.06 | 5 | -6.80 ± 0.05 | 5 |  |  | 1.3 |  | 6.2 |  | 4.6 |
| perphenazine | -6.00 ± 0.06 | 6 | -7.16 ± 0.05 | 6 | -6.83 ± 0.04 | 5 |  |  | 14.5 |  | 6.8 | 2.1 |  |
| ARC 239 | -5.99 ± 0.06 | 5 | -7.32 ± 0.14 | 6 | -7.25 ± 0.14 | 5 |  |  | 21.4 |  | 18.2 | 1.2 |  |
| sertindole | -5.95 ± 0.06 | 5 | -5.81 ± 0.07 | 5 | -6.17 ± 0.03 | 5 |  | 1.4 |  |  | 1.7 |  | 2.3 |
| flupenthixol | -6.10 ± 0.12 | 5 | -6.28 ± 0.13 | 5 | -6.88 ± 0.14 | 5 |  |  | 1.5 |  | 6.0 |  | 4.0 |
| imiloxan | -5.88 ± 0.03 | 6 | -6.48 ± 0.05 | 6 | -6.27 ± 0.03 | 6 |  |  | 4.0 |  | 2.5 | 1.6 |  |
| amitriptyline | -5.86 ± 0.05^app^ | 5 | -7.12 ± 0.05 | 5 | -6.67 ± 0.09 | 5 |  |  | 18.2 |  | 6.5 | 2.8 |  |
| clozapine | -5.86 ± 0.08^app^ | 5 | -6.20 ± 0.05 | 5 | -6.87 ± 0.08 | 5 |  |  | 2.2 |  | 10.2 |  | 4.7 |
| quetiapine | -5.81 ± 0.08 | 5 | -6.72 ± 0.08 | 5 | -6.66 ± 0.03 | 5 |  |  | 8.1 |  | 7.1 | 1.1 |  |
| bucindolol | -5.81 ± 0.05 | 5 | -5.63 ± 0.06 | 5 | -5.95 ± 0.04 | 5 |  | 1.5 |  |  | 1.4 |  | 2.1 |
| dibenamine | -5.80 ± 0.06 | 10 | -6.43 ± 0.06  -4.64 ± 0.07  60.9 ± 3.4% site 1 | 10 | -6.18 ± 0.05 | 10 |  |  | 4.3 |  | 2.4 | 1.8 |  |
| prochlorperazine | -5.78 ± 0.02^app^ | 6 | -6.46 ± 0.11 | 6 | -6.31 ± 0.09 | 6 |  |  | 4.8 |  | 3.4 | 1.4 |  |
| pimozide | -5.76 ± 0.12^ep^ | 5 | -6.30 ± 0.10 | 5 | -6.84 ± 0.05 | 5 |  |  | 3.5 |  | 12.0 |  | 3.5 |
| phenoxybenzamine | -5.72 ± 0.10 | 10 | -6.44 ± 0.11  -4.89 ± 0.08  51.4 ± 3.3% site 1 | 10 | -6.41 ± 0.11  -4.71 ± 0.13  74.1 ± 4.1% site 1 | 10 |  |  | 5.2 |  | 4.9 | 1.1 |  |
| clomipramine | -5.71 ± 0.07^app^ | 5 | -6.10 ± 0.13 | 5 | -5.80 ± 0.02^app^ | 5 |  |  | 2.5 |  | 1.2 | 2.0 |  |
| doxepin | -5.69 ± 0.12 | 5 | -6.67 ± 0.05 | 5 | -6.04 ± 0.07 | 5 |  |  | 9.5 |  | 2.2 | 4.3 |  |
| trimipramine | -5.67 ± 0.03 | 5 | -6.22 ± 0.05 | 5 | -6.37 ± 0.03 | 5 |  |  | 3.5 |  | 5.0 |  | 1.4 |
| sertraline | -5.67 ± 0.07^app^ | 6 | -5.62 ± 0.11^app^ | 6 | -5.64 ± 0.05^app^ | 6 |  | 1.1 |  | 1.1 |  | 1.0 | |
| chlorpromazine | -5.65 ± 0.13^app^ | 6 | -6.60 ± 0.12 | 6 | -5.93 ± 0.11 | 6 |  |  | 8.9 |  | 1.9 | 4.7 |  |
| nortriptyline | -5.65 ± 0.05 | 5 | -6.38 ± 0.02 | 5 | -6.19 ± 0.08 | 5 |  |  | 5.4 |  | 3.5 | 1.5 |  |
| vortioxetine | -5.63 ± 0.06^app^ | 5 | -5.32 ± 0.04^app^ | 6 | -5.84 ± 0.05 | 6 |  | 2.0 |  |  | 1.6 |  | 3.3 |
| trifluoperazine | -5.60 ± 0.05 | 5 | -6.22 ± 0.12 | 5 | -6.20 ± 0.06 | 5 |  |  | 4.2 |  | 4.0 | 1.0 | |
| olanzapine | -5.59 ± 0.05 | 5 | -5.47 ± 0.06 | 5 | -5.86 ± 0.02 | 5 |  | 1.3 |  |  | 1.9 |  | 2.5 |
| PF3774076 | -5.59 ± 0.04 | 6 | IC_50_>-4 | 6 | -5.29 ± 0.09 | 6 |  |  |  | 2.0 |  |  |  |
| promethazine | -5.58 ± 0.07 | 5 | -6.25 ± 0.06 | 5 | -5.54 ± 0.05 | 5 |  |  | 4.7 | 1.1 |  | 5.1 |  |
| cyanopindolol | -5.56 ± 0.10 | 5 | -4.82 ± 0.10^app^ | 5 | -6.15 ± 0.07 | 5 |  | 5.5 |  |  | 3.9 |  | 21.4 |
| alfuzosin | -5.56 ± 0.04 | 5 | -4.62 ± 0.05 | 5 | -6.14 ± 0.04 | 5 |  | 8.7 |  |  | 3.8 |  | 33.1 |
| Rec15-2615 | -5.53 ± 0.12^app^ | 6 | IC_50_>-4.5 | 6 | -6.56 ± 0.13 | 6 |  |  |  |  | 10.7 |  |  |
| urapidil | -5.49 ± 0.05 | 5 | -5.78 ± 0.08 | 5 | -6.34 ± 0.05 | 5 |  |  | 1.9 |  | 7.1 |  | 3.6 |
| silodosin | -5.49 ± 0.06^app^ | 6 | IC_50_>-5 | 6 | -6.12 ± 0.06^app^ | 6 |  |  |  |  | 4.3 |  |  |
| duloxetine | -5.43 ± 0.06 | 5 | -5.31 ± 0.09 | 5 | -5.67 ± 0.06 | 5 |  | 1.3 |  |  | 1.7 |  | 2.3 |
| haloperidol | -5.38 ± 0.06 | 5 | -5.53 ± 0.10 | 5 | -5.77 ± 0.05 | 5 |  |  | 1.4 |  | 2.5 |  | 1.7 |
| doxazosin | -5.35 ± 0.04 | 6 | -4.74 ± 0.07^app^ | 6 | -6.24 ± 0.02 | 6 |  | 4.1 |  |  | 7.8 |  | 31.6 |
| prazosin | -5.33 ± 0.05 | 6 | -6.17 ± 0.05 | 6 | -6.59 ± 0.04 | 6 |  |  | 6.9 |  | 18.2 |  | 2.6 |
| BMY7378 | -5.30 ± 0.03 | 5 | -4.98 ± 0.09^app^ | 5 | -6.26 ± 0.01 | 5 |  | 2.1 |  |  | 9.1 |  | 19.1 |
| norclomipramine | -5.29 ± 0.09^app^ | 6 | -5.74 ± 0.04^app^ | 6 | -5.80 ± 0.07^app^ | 6 |  |  | 2.8 |  | 3.2 |  | 1.1 |
| JP1302 | -5.29 ± 0.04 | 5 | -5.11 ± 0.05 | 5 | -6.92 ± 0.13 | 5 |  | 1.5 |  |  | 42.7 |  | 64.6 |
| imipramine | -5.25 ± 0.04 | 5 | -6.36 ± 0.08 | 5 | -5.89 ± 0.03 | 5 |  |  | 12.9 |  | 4.4 | 3.0 |  |
| terazosin | -5.18 ± 0.03 | 5 | -6.08 ± 0.05 | 5 | -6.27 ± 0.08 | 5 |  |  | 7.9 |  | 12.3 |  | 1.5 |
| 5-methyl-urapidil | -5.18 ± 0.05 | 5 | -5.17 ± 0.05 | 5 | -5.81 ± 0.07 | 5 |  | 1.0 | |  | 4.3 |  | 4.4 |
| dosulepin | -5.16 ± 0.06 | 5 | -6.20 ± 0.06 | 5 | -5.63 ± 0.11 | 5 |  |  | 11.0 |  | 3.0 | 3.7 |  |
| indoramin | -5.13 ± 0.03^app^ | 6 | -5.46 ± 0.05 | 6 | -5.80 ± 0.05 | 6 |  |  | 2.1 |  | 4.7 |  | 2.2 |
| amisulpiride | -5.11 ± 0.09^app^ | 5 | -4.69 ± 0.13^app^ | 5 | -5.57 ± 0.07 | 5 |  | 2.6 |  |  | 2.9 |  | 7.6 |
| domperidone | -5.09 ± 0.06^app^ | 6 | -5.29 ± 0.07 | 6 | -5.78 ± 0.08 | 6 |  |  | 1.6 |  | 4.9 |  | 3.1 |
| desipramine | -5.04 ± 0.06 | 5 | -5.78 ± 0.04 | 5 | -5.52 ± 0.03 | 5 |  |  | 5.5 |  | 3.0 | 1.8 |  |
| ICI 118551 | -5.03 ± 0.03 | 5 | IC_50_>-4 | 5 | -5.05 ± 0.04 | 5 |  |  |  | 1.0 | |  |  |
| cyclazosin | -5.00 ± 0.03 | 5 | -5.35 ± 0.13 | 5 | -6.18 ± 0.02 | 5 |  |  | 2.2 |  | 15.1 |  | 6.8 |
| protriptyline | -5.00 ± 0.05 | 5 | -5.39 ± 0.13 | 5 | -5.26 ± 0.07 | 5 |  |  | 2.5 |  | 1.8 | 1.3 |  |
| lofepramine | -4.86 ± 0.04^app^ | 5 | -5.60 ± 0.08 | 5 | -5.28 ± 0.06 | 5 |  |  | 5.5 |  | 2.6 | 2.1 |  |
| SDZ 21009 | -4.86 ± 0.07^app^ | 6 | IC_50_>-4 | 6 | IC_50_>-4.5 | 6 |  |  |  |  |  |  |  |
| propranolol | -4.85 ± 0.02 | 5 | IC50>-4 | 5 | -4.71 ± 0.06 | 5 |  |  |  | 1.4 |  |  |  |
| fluvoxamine | -4.81 ± 0.04^app^ | 6 | -4.37 ± 0.08^app^ | 5 | -4.82 ± 0.07^app^ | 6 |  | 2.8 |  | 1.0 | |  | 2.8 |
| fluoxetine | -4.70 ± 0.10^app^ | 5 | -4.99 ± 0.03 | 5 | -4.79 ± 0.07^app^ | 5 |  |  | 1.9 |  | 1.2 | 1.6 |  |
| AH11110A | -4.70 ± 0.04^app^ | 5 | IC_50_>-4 | 5 | -4.86 ± 0.03^app^ | 5 |  |  |  |  | 1.4 |  |  |
| carazolol | -4.66 ± 0.06^app^ | 6 | IC_50_>-4 | 6 | -4.66 ± 0.05^app^ | 6 |  |  |  | 1.0 | |  |  |
| labetolol | -4.62 ± 0.07^app^ | 5 | -4.71 ± 0.08^app^ | 5 | -5.27 ± 0.04 | 5 |  |  | 1.2 |  | 4.5 |  | 3.6 |
| sulpiride | -4.50 ± 0.02 | 5 | -4.37 ± 0.06 | 5 | -4.67 ± 0.07 | 5 |  | 1.3 |  |  | 1.5 |  | 2.0 |
| venlafaxime | -3.46 ± 0.03^app^ | 5 | IC_50_>-3 | 5 | -3.74 ± 0.11^app^ | 5 |  |  |  |  | 1.9 |  |  |
| 2-niguldipine | IC_50_>-5 | 5 | -5.48 ± 0.11 | 5 | -6.07 ± 0.11 | 5 |  |  |  |  |  |  | 3.9 |
| SNAP5089 | IC_50_>-5 | 5 | IC_50_>-5 | 5 | -5.65 ± 0.06 | 5 |  |  |  |  |  |  |  |
| paroxetine | IC_50_>-5 | 5 | IC_50_>-5 | 5 | IC_50_>-5 | 5 |  |  |  |  |  |  |  |
| citalopram | IC_50_>-4 | 5 | IC_50_>-4 | 5 | IC_50_>-4 | 5 |  |  |  |  |  |  |  |
| CGP20712A | IC_50_>-4 | 5 | IC_50_>-4 | 5 | -5.17 ± 0.03 | 5 |  |  |  |  |  |  |  |
| reboxetine | IC_50_>-4 | 5 | IC_50_>-4 | 5 | -4.56 ± 0.07^app^ | 4 |  |  |  |  |  |  |  |
| anisodamine | IC_50_>-3 | 5 | IC_50_>-3 | 5 | -3.56 ± 0.07^app^ | 5 |  |  |  |  |  |  |  |
| CGP12177 | IC_50_>-3 | 5 | No binding -3 | 5 | IC_50_>-3 | 5 |  |  |  |  |  |  |  |

^app^ = apparent affinity. The maximum concentration of competing ligand inhibited most but not all of specific binding. An IC_50_ was determined by extrapolating the curve assuming that all specific binding would be inhibited if a higher concentration of competing ligand were possible. Thus an apparent K_D_ was calculated.

^ep^ = early plateau, the competing ligand did not fully inhibit specific binding and the inhibition curve reached a plateau of maximal inhibition of binding. The specific binding inhibited by 3-MPPI was 75.6 ± 0.9% at α2A and 87.1 ± 1.5% at α2C and for pimozide was 79.1 ± 6.0% at α2A.
